# Comparative evaluation of screening approaches for antiviral monoclonal antibody combinations

**DOI:** 10.1128/jvi.00879-26

**Published:** 2026-09-02

**Authors:** Maria K. McClave, Samantha K. Marzi, Ekaterina E. Heldwein

**Affiliations:** 1 Department of Molecular Biology and Microbiology, Tufts University School of Medicine 12261 , Boston, Massachusetts, USA; 2 Graduate Program in Molecular Microbiology, Graduate School of Biomedical Sciences, Tufts University School of Medicine 12261 , Boston, Massachusetts, USA; 3 Graduate Program in Genetics, Molecular, and Cellular Biology, Graduate School of Biomedical Sciences, Tufts University School of Medicine 12261 , Boston, Massachusetts, USA; Dartmouth College Geisel School of Medicine, Hanover, New Hampshire, USA

**Keywords:** monoclonal antibody (mAb), neutralizing antibody, human cytomegalovirus (HCMV), antibody cocktail, antibody combination, checkerboard assay, DiaMOND, EMERALD

## Abstract

Monoclonal antibody (mAb) cocktails are increasingly used as antiviral therapeutics, but methods for evaluating mAb combination effects remain poorly standardized. To characterize interactions of recently isolated neutralizing mAbs targeting human cytomegalovirus glycoprotein B, we compared three screening approaches: checkerboard neutralization assays; DiaMOND, a high-throughput small-molecule interaction screening tool modified for mAb combinations; and EMERALD, a modified variable-ratio framework. As the most systematic approach, the checkerboard assay served as the baseline for subsequent comparisons. Most mAb combinations in checkerboard assays were classified as additive or antagonistic according to Loewe additivity-based analyses, regardless of whether the mAbs were competing or non-competing. Apparent synergy was observed primarily at saturating antibody concentrations. When adapted for mAb combinations, the DiaMOND framework produced interaction profiles that differed from checkerboard results under both concentration-based and dose-matched conditions. In contrast, EMERALD captured concentration-dependent interactions across variable mAb ratios and revealed asymmetric behavior, depending on which mAb was held constant. Together, these findings demonstrate that classifications of mAb interactions depend strongly on assay design, concentration range, and analytical framework. They further suggest that robust mAb synergy may be less common than often reported and highlight the need to evaluate antiviral mAb combinations across multiple experimental formats.

**IMPORTANCE:**

Monoclonal antibody cocktails are gaining foothold as an important strategy for the prevention and treatment of viral infections. Synergistic antibody combinations are often reported, but whether they represent true synergy is unclear because classifications of interactions depend on the experimental design and analytical methods. Moreover, there is no broadly accepted methodology for evaluating interactions among antibody combinations. As a result, it remains unclear whether common approaches for screening antibody combinations accurately capture biologically relevant interactions. Here, we compared four approaches for evaluating antibody combinations to help define best experimental and analytical practices for studying antiviral antibody interactions. Our findings demonstrate that antibody interactions are highly dependent on assay design, choice of antibody concentrations, and timing of antibody exposure, suggesting that synergy and antagonism are not fixed properties of antibody pairs. These findings may be broadly applicable to studies of antiviral antibody combinations across diverse viral systems.

## INTRODUCTION

Monoclonal antibody (mAb) combinations have emerged as an important therapeutic strategy for the treatment and prevention of viral infections, as antibody cocktails can broaden neutralization breadth, reduce the likelihood of viral escape, and improve therapeutic efficacy compared with single antibodies. Several successful antiviral antibody therapeutics, including those developed against the Ebola virus (1) and SARS-CoV-2 (reviewed in reference 2), have relied on combinations of mAbs targeting distinct epitopes and/or mechanisms of action. Despite this success, clinical antibody cocktails targeting human cytomegalovirus (HCMV) have demonstrated limited efficacy even when the individual component antibodies exhibited strong neutralizing activity (3, 4). These outcomes suggest that antibody neutralization potency alone may not be sufficient to predict an antibody cocktail’s performance and highlight the importance of thoroughly understanding how antibodies interact when combined.

**Fig 1:**
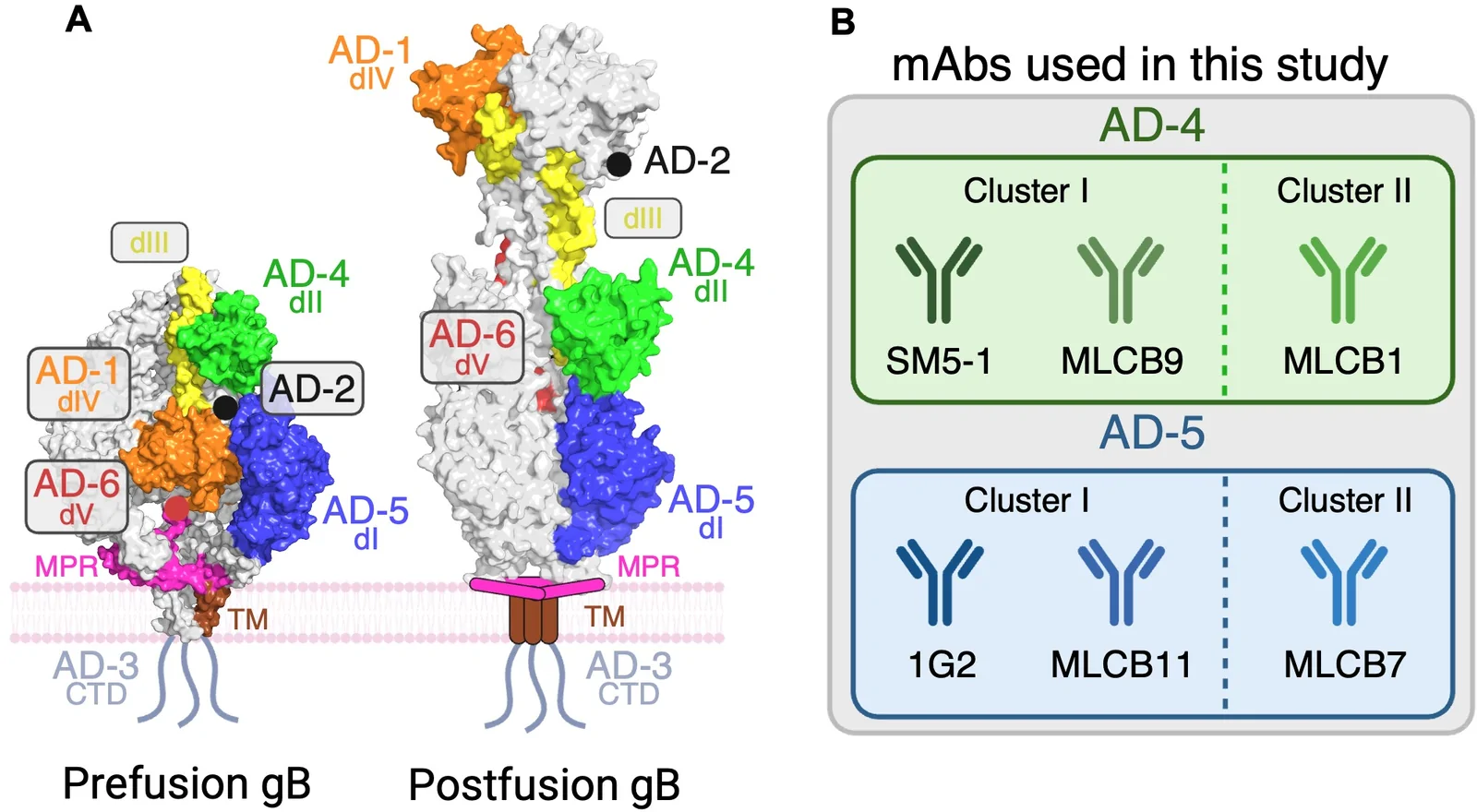
mAbs used in this study putatively bind to antigenic domains 4 and 5 on HCMV gB. (**A**) Surface models of prefusion (RCSB: 7KDP) and postfusion (RCSB: 5CXF) conformations of HCMV gB displaying one monomer colored and labeled with structural and antigenic domains (ADs). The terminal residue of AD-2 is represented by a black circle. The membrane proximal region (MPR), transmembrane domain (TM), and cytoplasmic domain (CTD) are represented in magenta, brown, and blue-gray, respectively. (**B**) mAbs used in this study as organized by putative AD binding site as determined in reference 5. Within each AD, mAbs are clustered according to previously established competition data, with antibodies within the same cluster displaying competition toward each other. Created in BioRender. McClave, M. (2026), https://BioRender.com/jj07ts9.

Recently, we reported the isolation of seven new neutralizing antibodies (nAbs) against a prefusion-stabilized HCMV glycoprotein B (gB) variant (5). HCMV gB is the viral fusogen that mediates the merger of the viral envelope and host cell membrane (reviewed in reference 6) (Fig. 1A). It is divided into six antigenic domains (reviewed in reference 7), termed AD-1–6 (Fig. 1A). Initial characterization of these antibodies revealed predominant binding to AD-4 and AD-5 (Fig. 1B) (5). Competition data provided further characterization of potential binding sites, and organizational clustering of competing and non-competing antibodies was established (Fig. 1B). We selected four of these seven antibodies, along with two previously published mAbs (8) (Fig. 1B), for use in downstream combination characterization experiments.

Antibody interactions can be broadly divided into three categories: synergy, additivity, and antagonism. The characterization of each type of interaction can be defined as the extent to which the combination of antibodies, each at half concentration, is able to reach baseline levels of neutralization. Additivity is defined as when, at half concentration of each antibody, the combination reaches baseline neutralization, and each antibody works as expected. Next, antagonism is defined as when the combination results in less than baseline neutralization, as the activity of one antibody is somehow disrupting the ability of the other antibody to act. Finally, synergy is defined as when the combination surpasses baseline level of neutralization, such that one antibody is enhancing the ability of the other to act and/or vice versa (9). Synergistic, additive, or antagonistic interactions can suggest whether antibodies act independently from one another, target overlapping functional processes, or interfere with one another directly.

To characterize these interactions, nAb combinations were initially evaluated using traditional checkerboard neutralization assays, which systematically assess combinatorial activity across a matrix of antibody concentrations (10, 11). While checkerboard assays provide a comprehensive assessment of antibody interactions, the large number of possible combinations among the seven isolated nAbs made this approach not feasible for broad screening applications. To address this, the DiaMOND (diagonal measurement of N-way drug interactions) (12) framework, generally used for small-molecule interactions, was adapted as a higher-throughput approach to evaluate antibody combinations. However, discrepancies between the checkerboard and DiaMOND results suggested that existing approaches may not fully capture the complexities of antibody-mediated neutralization. This prompted the development of EMERALD (equivalent-dose mapping via effect-based ratio analysis under locked dose conditions), based on a historical variable-ratio approach (13), designed to evaluate pairwise antibody interactions through the lens of one partner’s changes across variable concentrations.

To quantify these interactions and streamline comparisons across methods, we used the Loewe additivity model, which assumes that agents producing the same biological effect can be considered dose equivalent and predicts the expected response of a combination of the two agents as additive (14–16). Since all antibodies examined here target HCMV gB and inhibit the same biological process—viral entry—Loewe additivity provides an appropriate reference model for evaluating antibody interactions.

While the antibodies tested in this study may or may not have downstream clinical potential, our focus was primarily to highlight broader methodological challenges in antibody combination analysis. Anti-HCMV clinical antibody cocktails have demonstrated limited efficacy despite strong activity of individual component antibodies (3, 4), raising the possibility that current approaches for evaluating antibody combinations may overlook important antagonistic or concentration-dependent effects. Thus, this work represents an effort not only to characterize HCMV-specific antibody interactions but also to develop and optimize more rigorous experimental approaches to study antibody combinations and inform future therapeutic cocktail design.

## RESULTS

### Competing nAbs tested in a checkerboard assay primarily demonstrated additive or antagonistic interaction profiles

mAb combinations were initially evaluated for neutralization potency using a checkerboard assay format, in which pairs of mAbs were tested across a two-dimensional concentration matrix. This approach enables a comprehensive assessment of mAb–mAb interactions by measuring neutralization across all pairwise combinations, providing a detailed interaction landscape for each antibody pair. In our previous work, we tested the mAbs against three HCMV strains (AD169_GFP_, TR_GFP_, and TB40/4_GFP_) (5). Although some mAbs neutralized more than one strain, all mAbs used here neutralized AD169_GFP_ (5) (Table S1). Therefore, AD169_GFP_ was used for all assays described below.

Checkerboard assays were performed to both characterize interactions among newly isolated mAbs and identify combinations that may exhibit synergistic activity, with potential relevance for therapeutic development. mAbs were combined using concentration-matched titrations, consistent with standard practice in the HCMV field (10, 11, 17), in which each antibody is varied across the same concentration range regardless of potency differences. This design allows for direct comparison to prior studies and identification of interaction effects across a range of concentrations.

The first mAb combinations tested in a checkerboard viral neutralization assay were two pairwise combinations of competing mAbs, MLCB11/1G2 and MLCB9/SM5-1 (Fig. 1B; Fig. S1A and B and 2A and B). Since competing mAbs are expected to interact additively, they can be used to validate the assay methodology. Such an interaction profile is expected because sterically competing antibodies cannot, by definition, be bound to the same antigen simultaneously. Only one antibody would be sterically permitted to bind the antigen at any given time. Thus, half the surface antigens will be bound by one antibody and half by the other, assuming there are minimal differences in the binding kinetics. If each antibody is able to act in its normal capacity, competition need not lead to antagonistic interactions.

**Fig 2:**
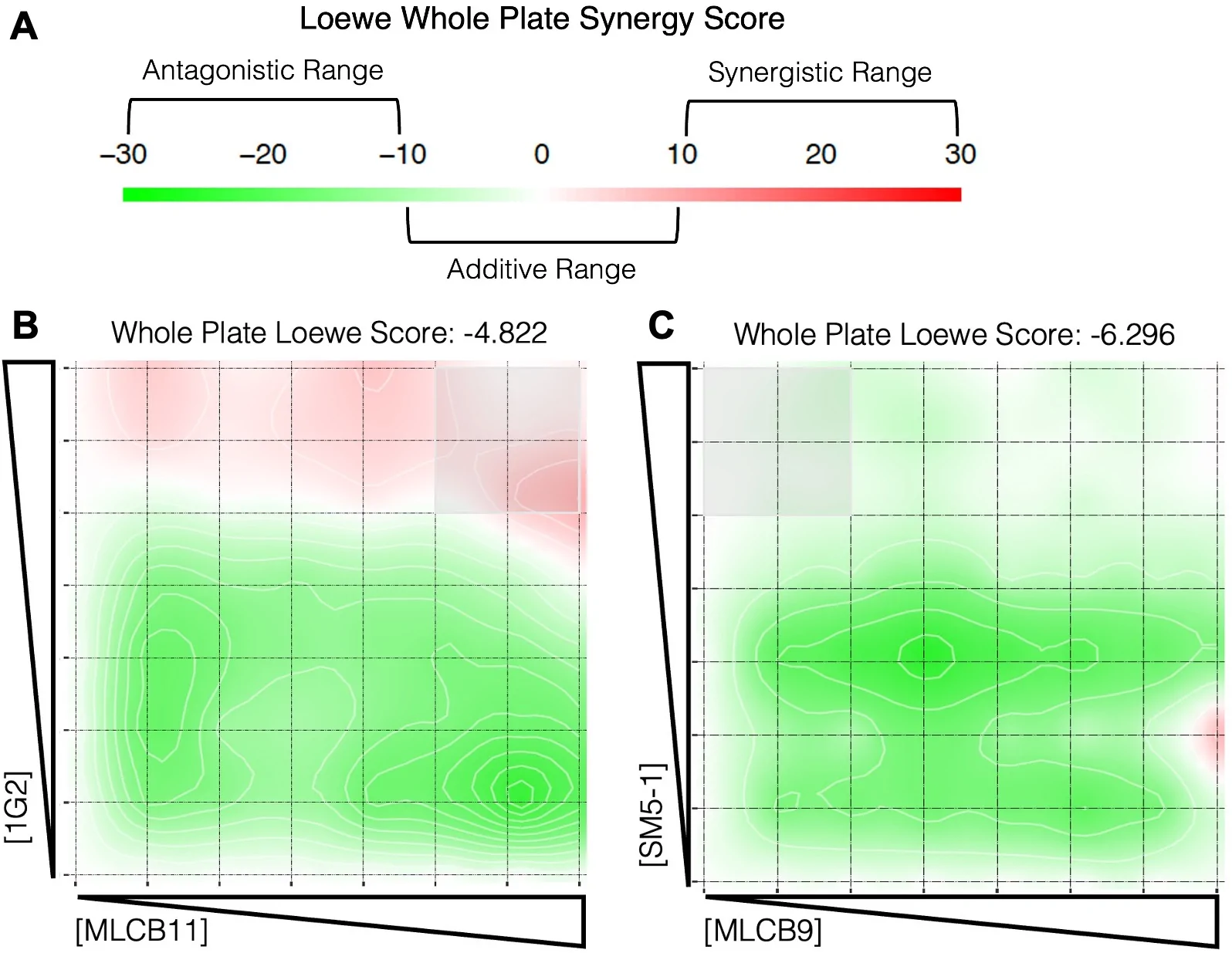
Competing nAbs tested in a checkerboard assay primarily demonstrated additive interaction profiles. (**A**) Interpretation guide for Loewe whole-plate synergy scores generated by SynergyFinder. Whole-plate Loewe scores between −10 and 10 are considered additive interactions; scores greater than 10 indicate synergistic interactions; and scores less than −10 indicate antagonistic interactions. Scores are visualized on a continuous color scale, where green indicates antagonism; white indicates additivity; and red indicates synergy. The color intensity reflects the magnitude of the interaction score. (**B **and **C**) Competing mAbs were tested in combination in a viral neutralization assay against HCMV AD169_GFP_. Results were entered into SynergyFinder to calculate the Loewe synergy score (top). Antibodies were combined at a 1:1 ratio and tested at matched concentrations across the checkerboard matrix (0–25 μg/mL). Triangular wedges along the axes indicate increasing concentrations of each antibody across the checkerboard matrix. The gray box indicates the highest area of synergy. Results are the average of three plate replicates. (**B**) MLCB11 and 1G2 whole-plate score of −4.822. (**C**) MLCB9 and SM5-1 whole-plate score of −6.296.

Data were then input into SynergyFinder to calculate an average Loewe synergy score for the whole plate. The Loewe additivity model compares the observed effect of a combination to the effect expected if the two antibodies simply contributed their individual activities without enhancing or interfering with one another. According to SynergyFinder classifications, a score between −10 and 10 indicates an additive effect; a score above 10 is synergistic; and a score below −10 indicates an antagonistic effect. These classifications are reflected visually as well, with green indicating antagonism, white indicating additivity, and red indicating synergy (Fig. 2A). In addition to providing a whole-plate score, SynergyFinder also highlights the “highest synergy area” in a gray 2 × 2 box on each plot (Fig. 2B and C). MLCB11 and 1G2 yielded a whole-plate Loewe synergy score of −4.822, consistent with an overall additive interaction across the checkerboard matrix (Fig. 2B). MLCB9 and SM5-1 yielded a whole-plate Loewe synergy score of −6.296, again consistent with an overall additive interaction across the checkerboard matrix (Fig. 2C). We note that the absolute neutralization potency measured for MLCB9 was lower than what we reported previously (5)(Table S1). Although the basis for this difference is unclear and may reflect inter-experimental variability, all mAbs analyzed in this study were evaluated under the same conditions such that the relative interaction analyses presented here are internally consistent.

In both cases, most of the dose–response landscape fell within the pale green to green range, indicating predominantly additive to antagonistic effects, respectively, throughout most concentration combinations tested (Fig. 2B and C). In the 1G2 and MLCB11 combination, a single area of light red signal was observed at a high concentration of 1G2, indicating modest, localized synergy. However, this localized synergy occurred near maximal concentration and may be due to saturation effects in the assay rather than genuine synergistic activity. Given that these pairwise combinations are known to compete for binding (5), the mildly antagonistic yet largely additive interaction profile observed across the matrix was consistent with their overlapping binding behavior, where only one mAb can act at a time.

### Non-competing nAbs tested in a checkerboard assay interacted largely antagonistically with synergy at high concentrations

**Fig 3:**
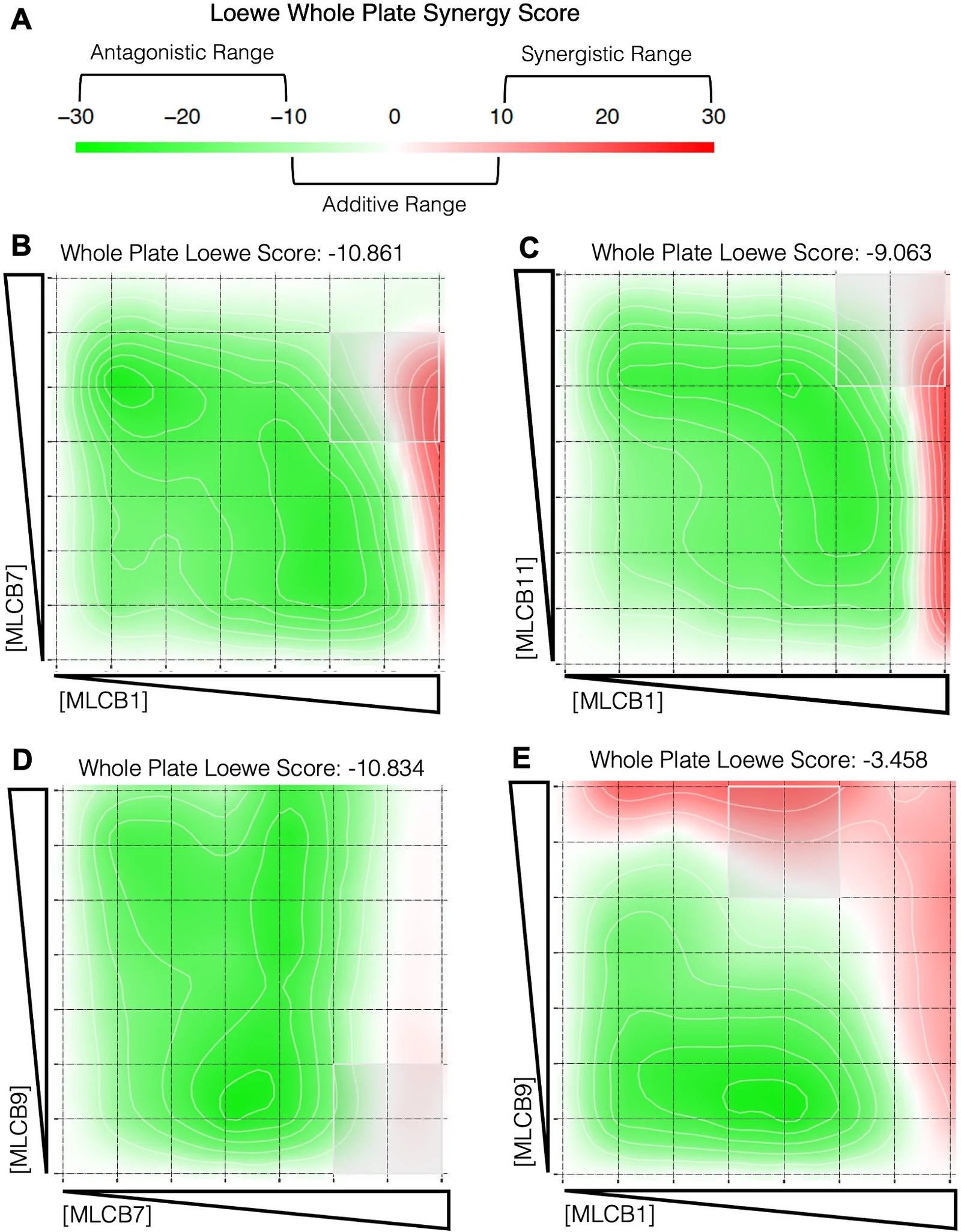
Non-competing nAbs tested in a checkerboard assay interact largely antagonistically, with possible synergy at saturating concentrations. (**A**) Interpretation guide for Loewe whole-plate synergy scores generated by SynergyFinder. Whole-plate Loewe scores between −10 and 10 are considered additive interactions; scores greater than 10 indicate synergistic interactions; and scores less than −10 indicate antagonistic interactions. Scores are visualized on a continuous color scale, where green indicates antagonism; white indicates additivity; and red indicates synergy. The intensity of the color reflects the magnitude of the interaction score. (**B–E**) Non-competing mAbs were tested in combination in a viral neutralization assay against HCMV AD169_GFP_. Results were entered into SynergyFinder to calculate the Loewe synergy score (top). Antibodies were combined at a 1:1 ratio and tested at matched concentrations across the checkerboard matrix (0–25 μg/mL). Triangular wedges along the axes indicate increasing concentrations of each antibody across the checkerboard matrix. The gray box indicates the highest area of synergy. Results are the average of three plate replicates. (**B**) MLCB1 and MLCB7 whole-plate score of −10.861. (**C**) MLCB1 and MLCB11 whole-plate score of −9.063. (**D**) MLCB7 and MLCB9 whole-plate score of −10.834. (**E**) MLCB1 and MLCB9 whole-plate score of −3.458.

To better understand the interactions between the mAbs, non-competing mAbs were tested in combination in checkerboard viral neutralization assays (Fig. 1B; Fig. S1C through F and S2D through F). Data were then input into SynergyFinder to calculate an average Loewe synergy score for the whole plate (Fig. 3A). SynergyFinder also identified the highest synergy area for each plate, as seen as a 2 × 2 gray box on each plot (Fig. 3B through E).

Combination testing of MLCB1 and MLCB7 yielded mildly antagonistic interactions across most of the checkerboard matrix (Loewe score of −10.861), with a small area of synergy at high concentrations of MLCB1 (Fig. 3B). A similar pattern was observed for MLCB1/MLCB11 (Fig. 3C), with a Loewe score of −9.063. MLCB7 and MLCB9 showed only an additive effect at high concentrations of MLCB7 and antagonism elsewhere on the matrix, corresponding to a Loewe synergy score of −10.834 (Fig. 3D). MLCB1 and MLCB9 interacted in an additive manner, judging by the Loewe score of −3.458. However, this overall score resulted from antagonism at lower concentrations and synergy at higher concentrations of both antibodies (Fig. 3E). In all cases, localized “synergy” occurred near maximal neutralization and may reflect saturation effects in the assay rather than robust synergistic activity.

### MLCB7 and MLCB9, added sequentially in a checkerboard assay, exhibited interaction patterns different from those observed when added simultaneously

**Fig 4:**
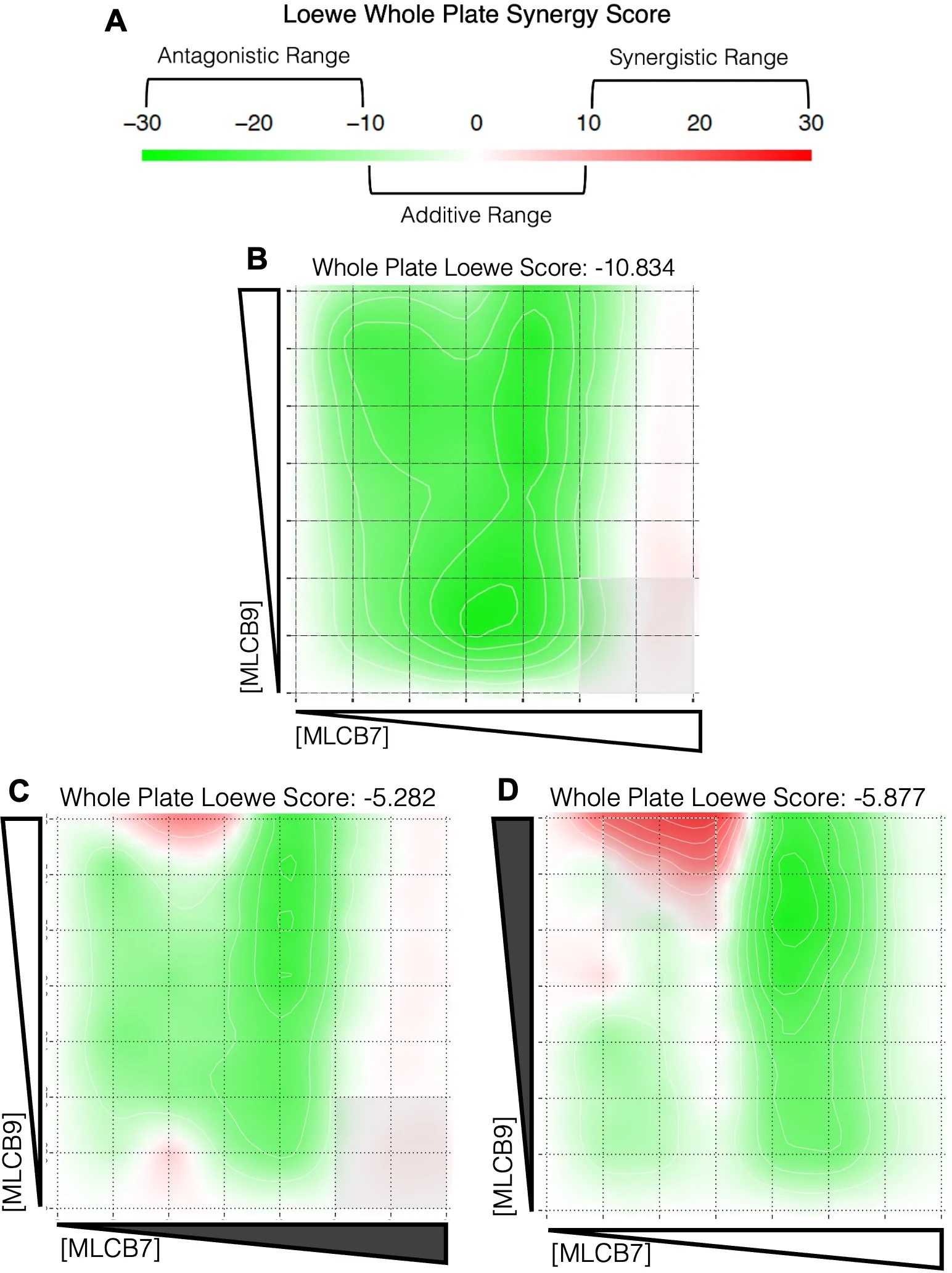
MLCB7 and MLCB9, added sequentially in a checkerboard assay, exhibit interaction patterns different from those observed when added simultaneously. (**A**) Interpretation guide for Loewe whole-plate synergy scores generated by SynergyFinder. Whole-plate Loewe scores between −10 and 10 are considered additive interactions; scores greater than 10 indicate synergistic interactions; and scores less than −10 indicate antagonistic interactions. Scores are visualized on a continuous color scale, where green indicates antagonism; white indicates additivity; and red indicates synergy. The color intensity reflects the magnitude of the interaction score. (**B**) Taken from Fig. 3D. MLCB7 and MLCB9 added simultaneously yield a whole-plate Loewe synergy score of −10.834. (**C** and **D**) mAbs were tested in combination in a sequential viral neutralization assay against HCMV AD169_GFP_. Results were entered into SynergyFinder to calculate the Loewe synergy score (top). Antibodies were combined at a 1:1 ratio and tested at matched concentrations across the checkerboard matrix (0–25 μg/mL). Triangular wedges along the axes indicate increasing concentrations of each antibody across the checkerboard matrix. The filled-in triangular wedge indicates the mAb added first. The gray box indicates the highest area of synergy. Results are the average of three plate replicates. (**C**) MLCB7 added before MLCB9 yields a whole-plate Loewe synergy score of −5.282. (**D**) MLCB9 added before MLCB7 yields a whole-plate Loewe synergy score of −5.877.

To better understand the mechanisms of MLCB7 and MLCB9, which were not predicted to be antagonistic based on their competition assay results (5) (Fig. 1B), mAbs were tested in a sequential checkerboard viral neutralization assay (Fig. S1G and H and S2G and H). Changes in mAb interactions across the whole matrix could be attributed to differences in neutralization mechanisms between the two mAbs. If pre-incubation with one mAb produced an interaction pattern similar to that seen with simultaneous mAb addition, but reversing the order resulted in a markedly different profile, this would suggest that the mAb added first exerts a dominant effect. In that case, the first mAb likely limits the ability of the second to function effectively unless the second mAb is given the opportunity to bind first. Data were then input into SynergyFinder to calculate an average Loewe synergy score for the whole plate (Fig. 4A). The highest synergy area was also identified and highlighted as a gray box on each plot (Fig. 4B through D).

When MLCB7 and MLCB9 were added simultaneously, their interaction profile was a mildly antagonistic interaction (Fig. 3D and 4B). In both cases of sequential addition, the whole-plate score indicated a much stronger additive interaction than that observed when the nAbs were added simultaneously (Fig. 4B through D). MLCB7 added before MLCB9 yielded a whole-plate Loewe score of −5.282 (Fig. 4C), similar to the score of MLCB9 added before MLCB7, −5.877 (Fig. 4D). Despite their similar whole-plate Loewe scores, the two sequential conditions exhibited distinct interaction landscapes. MLCB7 added before MLCB9 (Fig. 4C) more closely resembled the simultaneous-addition condition (Fig. 4B), with relatively consistent additive behavior across the plate. By comparison, MLCB9 added before MLCB7 (Fig. 4D) displayed a larger region of synergy that was counterbalanced by areas of increased antagonism, yielding a comparable average score despite greater local variation in interaction behavior. However, neither sequential addition fully replicated the simultaneous addition.

The reduced antagonism observed in the sequential neutralization assays suggests that the interaction between MLCB7 and MLCB9 may be influenced by binding kinetics during co-incubation. When both mAbs were added simultaneously, the interaction profile was broadly antagonistic, whereas sequential addition produced more additive interaction landscapes regardless of which antibody was added first. The similarity between the two sequential conditions suggests that neither mAb exerted a strongly dominant neutralization effect under these conditions. Instead, temporally separating antibody addition may reduce interference between the antibodies during neutralization. Differences in incubation time may have also contributed, as the second mAb in the sequential assay was exposed to virions for a shorter period than in the simultaneous condition. In addition, due to the delayed addition of the second mAb, the first mAb was exposed to a higher concentration of virions for the first hour of incubation than when the mAbs were added simultaneously.

### nAbs tested with concentration-based DiaMOND assays demonstrated increasing antagonism with increasing concentration

Combination neutralization was additionally evaluated using the DiaMOND assay format, which has previously been developed to assess interactions between small-molecule drug combinations (12). In this approach, antibody pairs were tested along a single fixed-ratio dose axis, rather than across a full checkerboard matrix, by combining the two antibodies at matched proportions and scaling the total dose across a dilution series. Per Loewe analysis standards, a combination index (CI) of 1 indicates an additive effect; a CI above 1 is antagonistic; and a CI below 1 indicates a synergistic effect. We expanded this range by accounting for the experimental error so that a CI between 0.9 and 1.1 indicated an additive effect; a CI above 1.1 indicated an antagonistic effect; and a CI below 0.9 indicated a synergistic effect. We mimicked the color scheme established by SynergyFinder to aid in visualization, with green indicating antagonism, white indicating additivity, and red indicating synergy. Although DiaMOND had been applied in drug combination studies, to our knowledge, it had not been used to evaluate antibody–antibody interactions.

Here, the DiaMOND framework was adapted to mAb combinations to assess whether it could serve as a higher-throughput alternative to traditional checkerboard assays for studying mAb interactions. Traditional DiaMOND assays use dose-based matching, based on a drug’s IC_50_ and IC_90_. However, since traditionally, mAb cocktail studies used concentration-based matching, both methods were evaluated to efficiently capture interaction effects while reducing the experimental burden of full matrix-based assays.

**Fig 5:**
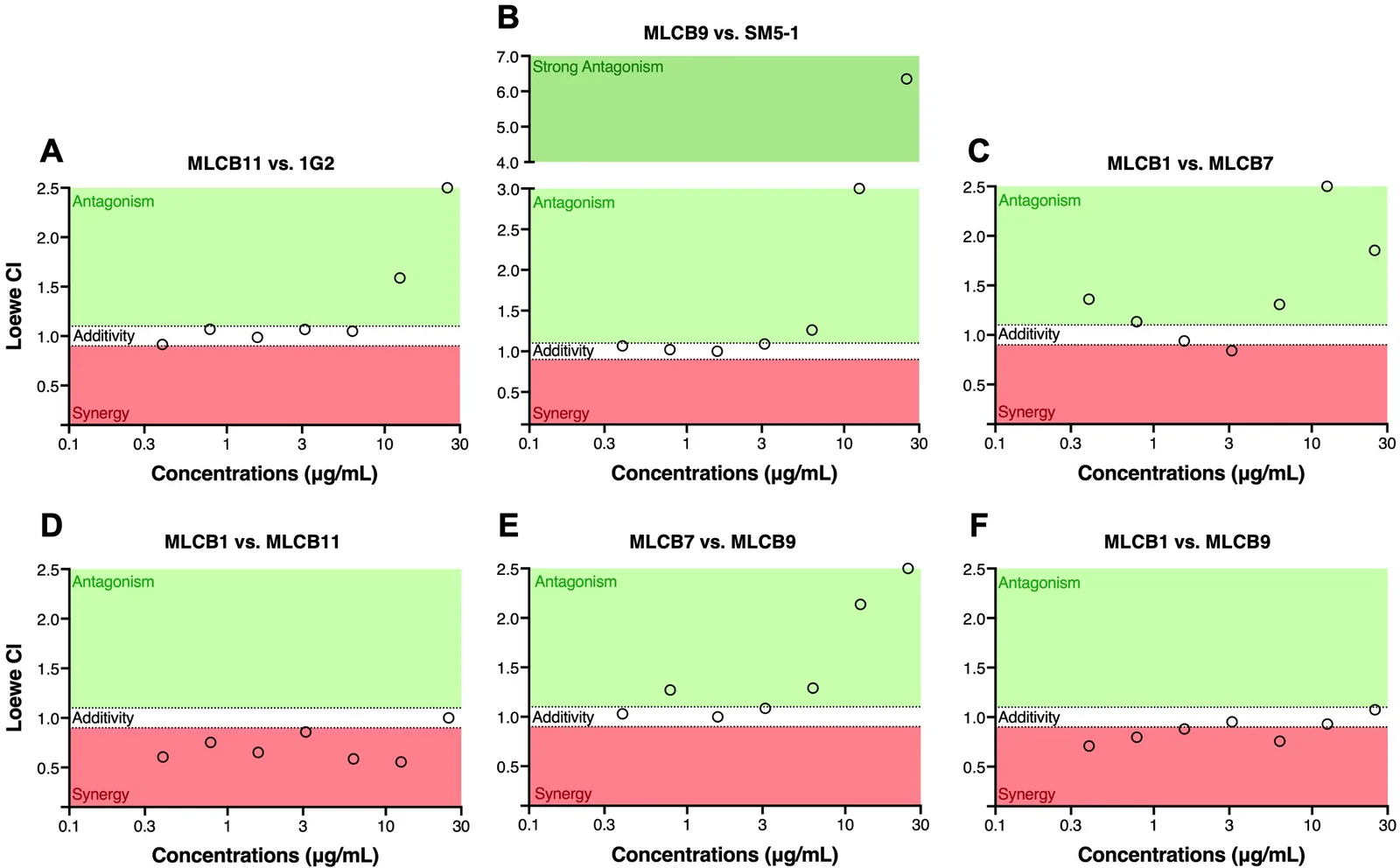
Concentration-based DiaMOND demonstrated increasing mAb/mAb antagonism with increasing concentration. (**A–F**) mAbs were tested in combination in a DiaMOND viral neutralization assay against HCMV AD169_GFP_. mAbs were combined 1:1 by concentration to mimic checkerboard assay conditions. The *x*-axis shows the concentration of each antibody in microgram per milliliter. The Loewe combination index (CI) was calculated for each concentration and plotted on the *y*-axis. A CI between 0.9 and 1.1 was considered an additive effect (white); a CI >1.1 was considered antagonistic (green); a CI <0.9 was considered synergistic (red); and a CI >3 was considered strongly antagonistic (dark green), as determined by experimental error. Antibody combinations are listed at the top of each graph. All graphs are the summary of three plate replicates.

Six mAb/mAb combinations that were assessed in checkerboard assays (Fig. 2 to 4) were evaluated. Generally, the mAb combinations showed additivity at lower concentrations, with distinct patterns at higher concentrations (Fig. 5; Fig. S3). In the case of MLCB11/1G2 (Fig. 5A) and MLCB9/SM5-1 (Fig. 5B), the two highest antibody doses indicated an antagonistic interaction. However, this variation could be due to saturation effects rather than true antagonism. MLCB1/MLCB7 showed mild antagonism at low concentrations, approaching additivity and mild synergy at central concentrations, with antagonism at higher concentrations (Fig. 5C). Both MLCB1/MLCB11 and MLCB1/MLCB9 combinations showed synergy at nearly every concentration (Fig. 5D and F). Finally, the MLCB7/MLCB9 combination showed a pattern similar to that of MLCB1/MLCB7, with additivity/mild antagonism at lower concentrations and antagonism at higher concentrations (Fig. 5E).

In summary, DiaMOND analysis revealed that most antibody combinations exhibited concentration-dependent interaction profiles, with additive effects predominating at lower concentrations and shifts toward antagonism or synergy at higher doses. While several combinations demonstrated largely additive or antagonistic behavior across the tested range, MLCB1/MLCB11 and MLCB1/MLCB9 consistently exhibited synergistic interactions. These findings demonstrate that the DiaMOND framework can be adapted to evaluate mAb–mAb interactions and identify concentration-dependent changes in interaction behavior using substantially fewer measurements than traditional checkerboard assays.

### mAbs tested with dose-based DiaMOND assays demonstrated peak antagonism at mid-range doses

**Fig 6:**
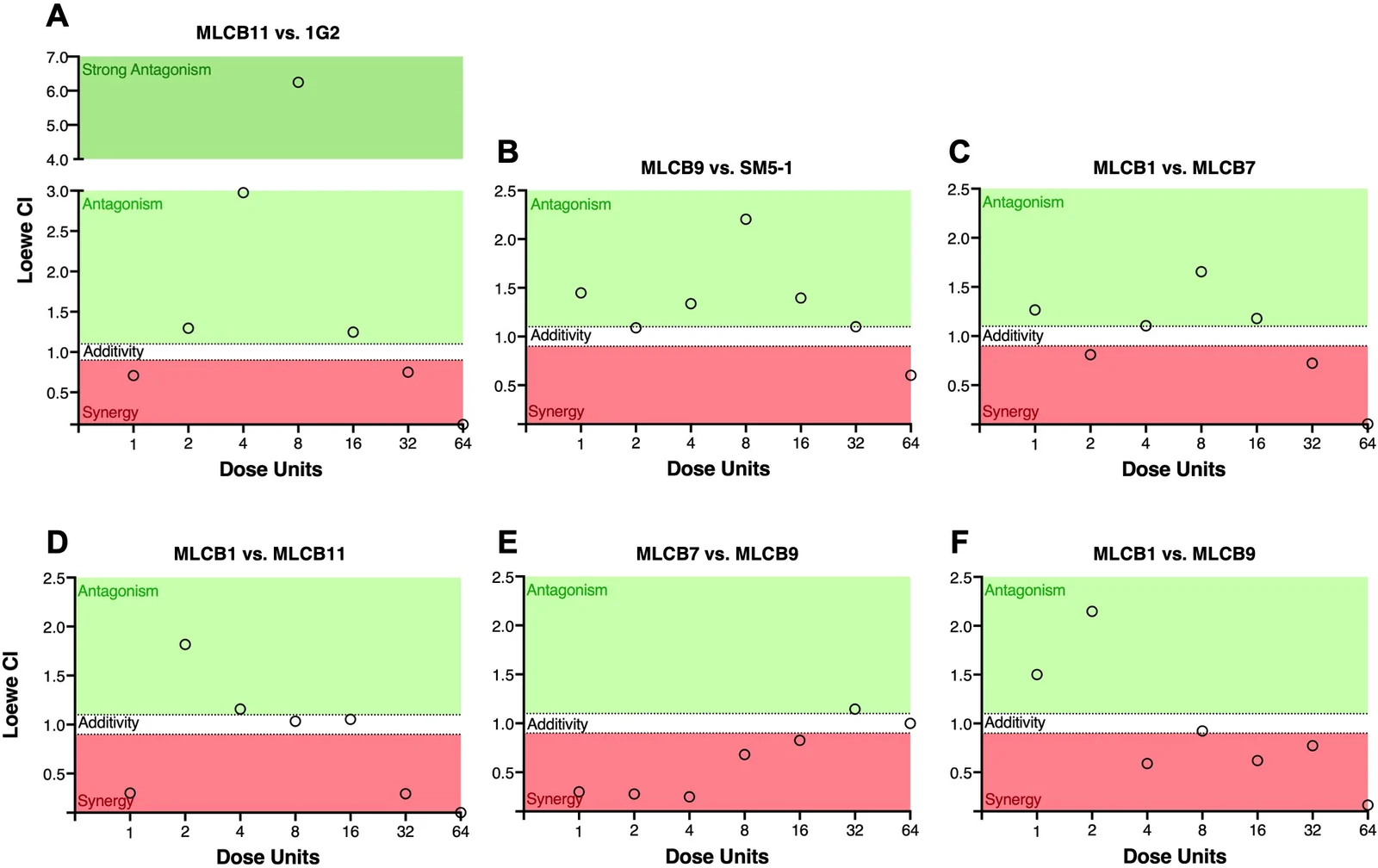
nAbs tested with dose-based DiaMOND assays demonstrated peak antagonism at mid-range concentrations. (**A-F**) mAbs were tested in combination in a DiaMOND viral neutralization assay against HCMV AD169_GFP_. mAbs were combined at a 1:1 dose ratio as calculated by individual titration curves. The *x*-axis shows the combined mAb doses. The Loewe combination index (CI) was calculated for each concentration and plotted on the *y*-axis. A CI between 0.9 and 1.1 was considered an additive effect (white); a CI >1.1 was considered antagonistic (green); a CI <0.9 was considered synergistic (red); and a CI >3 was considered strongly antagonistic (dark green), as determined by experimental error. Antibody combinations are listed at the top of each graph. All graphs are the summary of three plate replicates.

In almost all cases, the dose-based DiaMOND assays showed initial synergy at low doses, an increase in CI to antagonism at mid-range doses, followed by a return to synergy at high doses, forming a triangular shape (Fig. 6; Fig. S4). MLCB11/1G2 followed this pattern to an extreme, with the middle dose reaching a CI of ~6.5, indicating strong antagonism (Fig. 6A). MLCB9/SM5-1 showed an antagonistic interaction at the first dose, then followed a similar triangular interaction pattern to MLCB11/1G2, though with less extreme variation, as did MLCB1/MLCB7. (Fig. 6B and C). MLCB1/MLCB11 follows an elongated triangular shape, as the antagonistic peak occurs at the second dose and then slowly decreases through the additivity range (Fig. 6D). By contrast, MLCB7/MLCB9 did not follow this pattern; instead, their resulting interaction pattern begins in synergy and shifts to mild antagonism as dose increases, graphically resembling a sigmoidal curve (Fig. 6E). Finally, MLCB1 and MLCB9 also did not follow this general pattern, starting with antagonism at the lowest two doses and then transitioning to synergy at the remaining five doses (Fig. 6F).

In summary, dose-matched DiaMOND analysis revealed a pronounced dose-dependent variation in antibody interaction behavior. Most combinations exhibited non-linear changes in CI across the dose range, frequently transitioning between synergistic, additive, and antagonistic interaction states. While several antibody pairs displayed a characteristic triangular interaction profile, others followed distinct patterns, indicating that interaction behavior was highly dependent on both antibody identity and dose. These findings suggest that interaction classifications derived from dose-matched DiaMOND analyses are not fixed properties of antibody pairs but can vary substantially across the concentration range tested.

### mAbs tested using EMERALD assays demonstrated a wide range of interaction profiles

Combination neutralization was additionally evaluated using a variable-ratio assay format, in which mAb pairs were tested along a single-dose axis rather than across a full checkerboard matrix. This approach was based on a protocol described by Zwick et al. (13), in which HIV monoclonal antibody combinations were assessed by holding one antibody at its IC_50_ while titrating the second antibody across a full concentration range. In that study, combination effects were inferred from comparing resulting IC_50_ values to those of the individual antibodies. However, this approach provides only a coarse estimate of interaction effects and lacks the resolution to capture variation across the full neutralization curve.

Here, this framework was expanded by applying the Loewe additivity CI method to quantify interaction effects across the full titration series, rather than relying on a single summary metric. Per Loewe analysis standards, a CI of 1 indicates an additive effect; a CI above 1 is antagonistic; and a CI below 1 indicates a synergistic effect. We expanded this range by accounting for the experimental error so that a CI between 0.9 and 1.1 indicated an additive effect; a CI above 1.1 indicated an antagonistic effect; and a CI below 0.9 indicated a synergistic effect. Green was used to indicate antagonism, white to indicate additivity, and red to indicate synergy, in accordance with SynergyFinder classifications. This modification provides a more continuous and quantitative assessment of antibody interactions and forms the basis of the approach referred to here as EMERALD. This approach was explored in part to develop a higher-throughput method for screening antibody combinations, providing an alternative to more resource-intensive checkerboard assays while retaining the ability to detect synergistic or antagonistic interactions.

**Fig 7:**
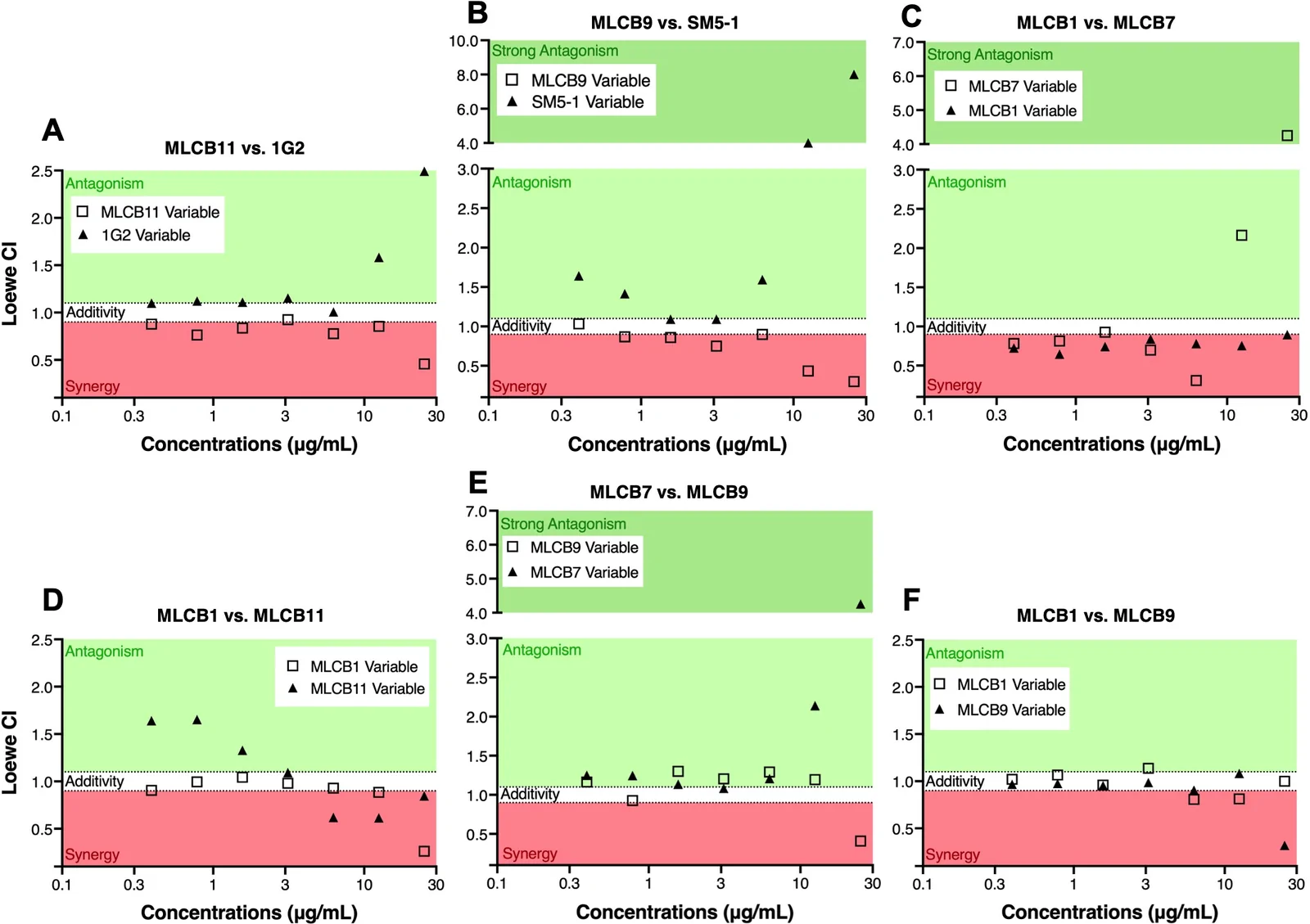
mAbs tested with EMERALD assays demonstrated a wide range of interaction profiles. (**A-F**) mAbs were tested in combination in an EMERALD viral neutralization assay against HCMV AD169_GFP_. One mAb was held constant at its IC_50_, while the second was varied across a range of concentrations (0–25 μg/mL). The filled triangles or open squares describe which mAb is being varied across the concentrations indicated on the *x*-axis in microgram per milliliter. The Loewe combination index (CI) was calculated for each concentration and plotted on the *y*-axis: 0.9 < CI < 1.1 indicated an additive effect (white); a CI >1.1 was considered antagonistic (green); a CI <0.9 was considered synergistic (red); and a CI >3 was considered strongly antagonistic (dark green), as determined by experimental error. Antibody combinations are listed at the top of each graph. All graphs are the summary of three plate replicates.

The interaction pattern for each of the two mAbs in the EMERALD assay varied, depending on which mAb was held constant. MLCB11 and 1G2 both started in an additive manner, but at higher concentrations, MLCB11 dipped into synergy when varied, whereas 1G2 showed antagonism (Fig. 7A). A similar pattern was observed with MLCB9 and SM5-1, although SM5-1 exhibited greater antagonism at lower doses (Fig. 7B). MLCB1 and MLCB7 showed mild synergy at nearly every concentration, except for when MLCB7 was at higher concentrations, where the mAbs showed antagonism (Fig. 7C). MLCB1 and MLCB11 showed very different interaction patterns, depending on which mAb was varied across a concentration gradient (Fig. 7D). When MLCB1 was varied, the mAbs were additive up to the highest dose, at which point they reached mild synergy or apparent synergy due to saturation (Fig. 7D). However, when MLCB11 was varied, the mAbs showed antagonism at low concentrations, dipping into synergy at higher concentrations in a graphically sigmoidal pattern (Fig. 7D). MLCB7 and MLCB9 primarily showed mild antagonism for almost every concentration, except for large deviations into strong antagonism or synergy at the highest concentrations, most likely due to saturation (Fig. 7E). MLCB1 and MLCB9 were additive across nearly all concentrations, except at the highest tested concentration of MLCB9 where they exhibited synergy (Fig. 7F).

In summary, EMERALD analysis revealed a broad range of additive, antagonistic, and synergistic interaction profiles across the antibody combinations tested. Antibody pairs such as MLCB11/1G2 and MLCB1/MLCB11 exhibited markedly different interaction patterns, depending on which antibody was held constant and which was titrated, indicating that interaction behavior was not necessarily symmetric within a given pair. By incorporating Loewe-based combination index analysis across the full concentration range, EMERALD provided a more quantitative assessment of antibody interactions than traditional variable-ratio approaches, which typically rely on a single summary metric. This increased resolution enabled visualization of concentration-dependent shifts in interaction behavior, revealing transitions among additive, antagonistic, and synergistic states that would not be apparent from endpoint measurements alone.

## DISCUSSION

### Saturation-driven effects may mimic antibody synergy

Across multiple checkerboard interaction matrices, localized regions of apparent synergy were observed primarily at the highest antibody concentrations tested (Fig. 2 to 4). However, these synergistic regions consistently occurred near maximal neutralization, suggesting that the observed effects may reflect saturation behavior rather than true cooperative biological interactions. In contrast, most of the checkerboard landscapes demonstrated additive or antagonistic interaction profiles across lower and intermediate concentrations, suggesting that strong synergistic activity was not a dominant feature of the antibody combinations tested.

This distinction is important because antibody interaction analyses are highly dependent on both the experimental design and the mathematical model used to interpret the resulting data. In recent years, an increasing number of HCMV antibody cocktail studies have reported synergistic interactions, particularly in the context of antiviral therapeutic development (10, 11, 17). However, several commonly used analytical models, including Bliss Independence and ZIP, may overestimate synergy when antibody effects are not truly independent (18, 19). SynergyFinder itself notes that different analytical methods can yield substantially different interaction classifications and recommends using multiple models to validate interaction interpretations (20). In the case of this study, Loewe was used exclusively to better compare results across the three different assays and to account for the lack of independence (i.e., mAbs targeting the same antigen).

This consideration may be particularly relevant for mAb cocktails. Unlike small-molecule drugs, antibodies frequently target the same viral glycoproteins, compete sterically, alter epitope accessibility, or converge on overlapping downstream neutralization mechanisms. Under these conditions, the assumption of independent action underlying Bliss-based approaches will likely not hold. Previous studies have noted that Bliss-based analyses can artificially inflate apparent synergy when effects saturate or when one antibody dominates the neutralization response (18, 21). Furthermore, because neutralization assays inherently approach an upper detection limit, combinations tested near maximal occupancy may generate apparent interaction effects that reflect compression of the response curve rather than true enhancement of antiviral activity, sometimes referred to as the “hook effect” (22).

The checkerboard results observed here support this interpretation. In nearly all cases, the strongest apparent synergy occurred only when at least one antibody was present at the highest concentration tested, while lower and intermediate concentrations yielded largely additive or antagonistic interactions (Fig. 2 to 4). Similar patterns have been reported in small-molecule studies, where synergy was calculated primarily from localized regions at maximal concentrations rather than broad improvements across the interaction landscape (23). This study raised the possibility that the observed synergy may instead reflect saturation-driven phenomena or limitations of the analytical framework (23). Most interesting, perhaps, is that akin to our results using DiaMOND and EMERALD, the aforementioned study found that when the same compounds were tested in limited-concentration curves, the calculated interactions were not concordant with checkerboard results. They noted that a limitation of all synergy testing, for small molecules and antibodies alike, is quality control standards for what is considered synergy and “a lack of established correlations between *in vitro* results and clinical outcomes when combinations are used to treat patients” (23). This phenomenon has also been reviewed in the context of antimicrobials (24). Importantly, the apparent synergy observed at high concentrations likely reflects increased occupancy of epitopes rather than the non-specific addition of antibody. Accordingly, non-specific antibodies that do not interact with the same system (i.e., the same virus) would not be expected to produce the same effect.

These findings highlight the importance of considering antibody potency and method of analysis during experimental design. Traditional checkerboard assays in the HCMV field often employ concentration-matched titrations, in which antibodies are combined at identical concentrations regardless of potency differences (10, 11, 17). However, concentration matching does not necessarily match the biological effect. When one antibody is substantially more potent than the other, a concentration-matched design may effectively test one antibody near saturation, while the other remains within its functional threshold. Under these conditions, interaction measurements may become biased toward the dominant antibody and fail to adequately sample the biologically relevant response. Although this phenomenon has not been formally discussed with regard to antibody–antibody interactions, several studies have emphasized that isoeffective, or potency-normalized, dosing is required for meaningful small-molecule interaction analysis and that fixed-concentration designs can undersample relevant regions of the response surface (19, 21, 25). In other fields, primarily HIV mAb studies, dose-based matching is, in fact, utilized, likely contributing to the success of these mAbs (26, 27).

This limitation likely contributes to the differences in behavior observed among the checkerboard, DiaMOND, and EMERALD assays in the present study. Matching fixed-ratio concentrations incompletely accounted for potency differences between antibodies, whereas dose-based or variable-ratio approaches partially altered the resulting interaction landscapes. Together, these findings suggest that antibody interaction behavior is highly dependent on the concentration space sampled during the assay. As a result, conclusions regarding synergy or antagonism should not rely solely on single concentration ranges, fixed-ratio designs, or isolated high-dose interaction regions.

Overall, these data suggest that true antibody synergy may be less common than currently implied in the HCMV antibody literature, particularly when assessed using stringent additivity models such as Loewe and when interaction behavior is evaluated across an entire response surface rather than at isolated concentrations. Rather than existing as strictly synergistic or antagonistic pairs, many antibody combinations may instead transition between additive, antagonistic, and potentially synergistic states, depending on concentration, occupancy, and assay design. Future antibody cocktail studies may therefore benefit from incorporating multiple analytical frameworks, broader ratio sampling, and full interaction landscapes to more rigorously distinguish true biological synergy from saturation-associated artifacts.

To this end, when comparing the results across our assays, we averaged the data from the central five concentrations used in each assay (Table 1). This approach minimized the influence of saturation at the highest concentrations while avoiding increased variability near the lower limit of detection, thereby focusing the comparison on the dynamic range most representative of each antibody pair activity.

**TABLE 1:** Comparison of antibody interaction classifications across assay formats^*a*^

Antibody pair	Comp?	Checkerboard	Concentration DiaMOND	Dose-based DiaMOND	EMERALD
MLCB11/1G2	Yes	Additive	Additive	Antagonism	Additive
MLCB9/SM5-1	Yes	Additive	Additive	Antagonism	Variable
MLCB1/MLCB7	No	Antagonism	Mild antagonism	Variable	Mild synergy
MLCB1/MLCB11	No	Antagonism	Synergy	Additive	Additive
MLCB7/MLCB9	No	Antagonism	Mild antagonism	Synergy	Mild antagonism
MLCB1/MLCB9	No	Antagonism	Mild synergy	Mild synergy	Additive

^*a*^ Each mAb/mAb pair tested is listed, along with their competition results and a summary of the pair’s interaction profile for each method used. The summary was obtained by averaging the Loewe score of the middle five doses/concentrations for each pair to avoid averaging data from saturating doses/concentrations. In the case where interaction profiles varied greatly and would be misrepresented by averaging, the classification “variable” was used. All averages are based on data shown in Fig. 2 to 7. “Comp” stands for competing.

### DiaMOND may offer a higher-throughput technique for screening antibody combinations

Across the checkerboard assays, most antibody combinations demonstrated predominantly additive-to-antagonistic interaction profiles, regardless of whether the antibodies were classified as competing or non-competing (Fig. 2 to 4; Table 1). Whole-plate Loewe scores generally fell within the additive or antagonistic range, and most interaction matrices were characterized by broad regions of green signal corresponding to antagonism, with limited areas of additivity. In several non-competing combinations, including MLCB1/MLCB7, MLCB1/MLCB11, and MLCB1/MLCB9, localized regions of apparent synergy were observed only at high antibody concentrations and, therefore, could be due to saturation-driven effects rather than true synergy (Fig. 3B, C and E). Similarly, MLCB7 and MLCB9 exhibited broadly antagonistic interaction landscapes during simultaneous addition, despite being predicted to be non-competing by binding analyses (Fig. 3D; Table 1). Together, these findings indicate that most mAb combinations tested did not exhibit robust synergy across the checkerboard matrix but instead showed concentration-dependent shifts between antagonistic and additive interactions.

However, competing antibody pairs generally exhibited more uniform interaction landscapes characterized by overall additivity, whereas non-competing pairs displayed greater variability, ranging from strongly antagonistic interactions to localized pockets of synergy at saturating concentrations. One possible explanation is that competition for overlapping epitopes constrains the range of potential interactions between competing antibodies, resulting in relatively predictable occupancy-driven effects, where occupancy refers to the fraction of available epitopes bound by an antibody at a given concentration. In contrast, non-competing antibodies can bind simultaneously and may therefore influence one another through more complex mechanisms, including changes in glycoprotein conformation, epitope accessibility, steric interactions, or fusion dynamics, leading to a broader spectrum of antagonistic and synergistic behaviors.

When concentration-based DiaMOND assays were compared to checkerboard results, the resulting interaction profiles only occasionally recapitulated the broader checkerboard interaction landscapes (Fig. 5; Table 1). Several combinations that appeared predominantly antagonistic in checkerboard assays demonstrated apparent synergy across much of the DiaMOND axis. For example, both MLCB1/MLCB11 and MLCB1/MLCB9 showed widespread synergistic CI values in concentration-based DiaMOND assays despite exhibiting largely antagonistic checkerboard interaction matrices (Fig. 3C, E, 5D and F; Table 1). Likewise, MLCB1 and MLCB7 demonstrated shifting interaction profiles with hints of mild synergy across the DiaMOND axis that did not clearly reflect the predominantly antagonistic checkerboard landscape (Fig. 3B and 5C; Table 1). Although some combinations, such as MLCB7 and MLCB9, retained increasing antagonism at higher concentrations in both assay formats, the overall interaction trends remained inconsistent between the two approaches (Fig. 3D and 5E). Together, these findings suggest that concentration-based DiaMOND assays do not fully capture the broader interaction landscapes observed in checkerboard analyses. This can most easily be explained by differences in the methods of analysis. DiaMOND reduces the data to a smaller set of measurements and applies a slightly different analytical framework than what is used by SynergyFinder, which compares results across the whole board. As a result, even similar neutralization data may yield different interaction classifications because small deviations from additivity can be interpreted differently between methods.

Similarly, dose-based DiaMOND assays demonstrated interaction patterns that did not reflect the overall trends observed in checkerboard assays (Fig. 6; Table 1). In most cases, dose-based DiaMOND assays produced a characteristic triangular interaction pattern, consisting of initial synergy at low doses, increased antagonism or additivity at intermediate doses, and a return toward synergy at higher doses. For combinations such as MLCB11/1G2, MLCB9/SM5-1, and MLCB1/MLCB7, this triangular pattern more closely mirrored the checkerboard results, which similarly demonstrated predominantly additive or antagonistic interactions with localized shifts at higher concentrations, most likely due to saturation effects (Fig. 2B, C, 3B, and 6A through C; Table 1). However, the initial synergy observed at low doses did not correspond well with checkerboard interaction profiles. As with synergistic CI values observed at high doses, low-dose synergy in DiaMOND assays may also represent an assay-specific phenomenon rather than true synergy, further supporting our concern of lower limits of detection and the influence of saturation within these assays. Although this phenomenon has not previously been described, it may reflect limitations associated with adapting DiaMOND methodologies, originally developed for small-molecule drugs, to antibody–antibody interactions, as antibody–antibody interactions may introduce more complicated interaction landscapes than the method can accurately capture. Alternatively, it is possible that DiaMOND assays can capture synergy not seen in a checkerboard setup, a phenomenon which should be investigated further with additional combinations.

Notably, combinations with more distinct checkerboard interaction patterns, including MLCB7/MLCB9 and MLCB1/MLCB9, did not recapitulate checkerboard assay results in the dose-based DiaMOND format at all, as both combinations displayed stable synergy across the dose axis despite predominant results of antagonism in the checkerboard assay (Fig. 3D, E, 6E and F; Table 1). Together, these findings suggest dose-based DiaMOND assays still fail to capture the full interaction landscapes observed in matrix-based neutralization assays. However, comparing concentration-based checkerboards to dose-based DiaMOND is an inherent limitation in our analysis, as the concentration gradient differs between assays. It is possible that dose-based DiaMOND assays would align more readily with dose-based checkerboards. In any case, they still offer insight into interactions within the biologically relevant working ranges of the antibodies.

### EMERALD assays capture concentration- and direction-dependent antibody interaction profiles but do not recapitulate checkerboard results

Unlike checkerboard and DiaMOND assays, which combine antibodies at matched concentrations or matched inhibitory doses, EMERALD uses a variable-ratio design in which one antibody is held constant, while the second is titrated across a full concentration range. This approach was adapted from Zwick et al., who evaluated HIV-1 mAb combinations by holding one antibody at a weakly neutralizing concentration while titrating the second antibody, then inferred synergy through shifts in IC_50_ values (13). In the present study, this framework was expanded by calculating Loewe CI values across the full titration series, thereby quantifying interactions at each concentration rather than inferring them from a single IC_50_ shift (Fig. 7).

This modification is important because antibody combinations may not behave uniformly across the dose–response curve. A single IC_50_ comparison can indicate that a combination is, overall, more potent or less potent, yet it cannot distinguish whether that shift reflects synergy, additivity, antagonism, or a concentration-dependent effect. With the help of Loewe CI analysis, EMERALD provided a more nuanced view of each interaction, especially as compared to the traditional variable-ratio design, revealing that several combinations changed classification, depending on which antibody was held constant and which was varied (Fig. 7). This was particularly apparent for MLCB1 and MLCB11, where varying MLCB1 produced a largely additive profile, whereas varying MLCB11 produced antagonism at low concentrations followed by a shift toward synergy at higher concentrations (Fig. 7D).

A major advantage of EMERALD is that it avoids an important limitation of matched-dose designs; namely, it does not assume that two antibodies should be compared only at equivalent concentrations or equivalent inhibitory doses. Instead, it asks whether the activity of a titrated antibody changes in the presence of a fixed amount of a second antibody. This may be particularly useful for mAbs with different potencies, slopes, or mechanisms because clinically or biologically relevant combinations may not occur at perfectly matched ratios. However, the choice of fixed concentration is likely critical. Holding one antibody at its IC_50_ may be useful because it preserves dynamic range for detecting both enhanced and reduced activity, but it may underrepresent interactions that only emerge at higher neutralization thresholds. In contrast, holding one antibody at its IC_90_ could more closely approximate conditions with strong neutralizing pressure and may better account for potency differences between antibodies, but it also risks compressing the remaining dynamic range and making apparent interaction effects harder to interpret due to saturation of the antigen.

Thus, IC_50_- and IC_90_-based EMERALD assays may answer related but distinct questions. IC_50_-based EMERALD may be better suited for detecting whether one antibody broadly shifts the potency of another across the middle of the neutralization curve, while IC_90_-based EMERALD may be more informative for identifying interactions under high-occupancy or near-maximal neutralization conditions. Because neutralizing antibodies can differ not only in potency but also in curve slope and maximum neutralization capacity, neither fixed point is likely to be universally optimal. Rather, the fixed concentration should be selected based on the biological question being asked, and future adaptations of EMERALD may benefit from testing both IC_50_- and IC_90_-anchored conditions.

Compared with checkerboard assays, EMERALD was less comprehensive because it sampled only two variable-ratio axes rather than the full two-dimensional interaction landscape. As a result, it may have missed interaction regions that occur only at specific paired concentrations. However, compared with DiaMOND, EMERALD offers greater flexibility, as it is not restricted to a single fixed-ratio diagonal. This made direction-dependent interaction patterns visible, including cases where the observed CI profile varied, depending on which antibody was held constant (Fig. 7). Therefore, EMERALD may serve as a useful intermediate approach: less resource intensive than full checkerboard assays, but more informative than fixed-ratio DiaMOND screening for identifying concentration-dependent or asymmetric antibody interactions.

Overall, EMERALD did not reproduce checkerboard results, but it provided a more nuanced view of antibody interactions than approaches based solely on matched concentrations, matched doses, or IC_50_ shifts. The addition of Loewe CI analysis strengthened the original variable-ratio framework by converting qualitative curve shifts into concentration-resolved interaction classifications. These findings suggest that EMERALD may be particularly useful as a screening strategy for identifying antibody pairs that warrant full checkerboard follow-up, especially when antibody potency, dose ratio, or directionality may influence the observed interaction profile.

### Limitations of our study and considerations for the future of comprehensive antibody testing

Our study has several caveats. First, all experiments were conducted in fibroblasts with AD169_GFP_ and, therefore, captured only a small subset of potential antibody interactions. Hence, we cannot speculate on any mechanistic interactions due to this limited system. However, the purpose of this study was not to investigate the clinical relevance of our antibodies but instead to challenge the field standard of only using a single assay for therapeutic testing. In this context, our use of a minimal system strikingly showcases the significant differences in antibody interactions between similar assays, a point that may be less clear upon the introduction of additional variables. However, for downstream testing of therapeutic cocktail candidates, experiments should be performed across an array of cell types and viral strains to account for the wide trophism and strain diversity of HCMV.

Although no single method used in this study offers a perfect solution to the need in the HCMV field for a method for comprehensive antibody interaction analysis, we have identified attractions and drawbacks to each, which may aid in directing downstream studies, depending on their goal. Concentration-based checkerboards disregard differences in potency of antibodies tested and may contribute to inflated reported synergy due to saturation effects. However, these offered the broadest overview of an antibody interaction landscape. DiaMOND and EMERALD offer a faster, higher-throughput alternative to traditional assays, but both of these assays are, by design, limited in the range of combinations tested. Additionally, EMERALD could be used to investigate the dominant antibody mechanism within a pair.

What is clear from all the assays utilized in this study, however, is the need for potency normalization in future combination testing, as it may aid in reducing overreported synergy due to saturation effects and offer more physiologically relevant testing conditions. Potency normalization, whether based on Hill slopes, IC_50_ values, or IC_90_ values, focuses on interactions within the working ranges of each antibody and avoids complications due to disparities between IC_50_ values. Because these normalization approaches are derived from the individual dose–response curves of each antibody, the Hill slope influences the expected additive response. Consequently, potency matching at IC_50_ and IC_90_ is not necessarily expected to yield identical interaction classifications, as each represents a different region of the dose–response curve (28, 29). Therefore, both metrics could and should be used in parallel approaches.

As mentioned before, dose-based matching has been successful in the HIV field (26, 27), and therefore, we are encouraged by its potential in HCMV antibody studies. Expanding the range of dose-based combinations tested to a full checkerboard, rather than a simplified setup of DiaMOND and EMERALD, may be more informative, given our evidence that antibody interactions are very sensitive to concentration changes. While the checkerboard is more labor intensive, it may be necessary to begin to fully characterize antibody interactions in a manner that would be translatable for downstream therapeutic analysis. Perhaps, to increase throughput, DiaMOND or EMERALD can be utilized as screening tools, followed by more rigorous dose-based checkerboard analysis. Ultimately, our findings support the use of multiple complementary approaches to evaluate antibody interactions rather than relying on any single assay. Until it is clear which metrics best predict biological or clinical efficacy, integrating orthogonal methodologies will provide a more complete understanding of the context-dependent nature of antibody interactions and better inform the rational design of antibody cocktails.

## MATERIALS AND METHODS

### Cells

Human foreskin fibroblasts (HFFs) (Hs27, ATCC CRL-1634) were cultured in Dulbecco’s modified Eagle medium (DMEM) containing 10% fetal bovine serum (FBS), 1% penicillin–streptomycin, and 2 mM L-glutamine after being thawed from frozen storage. The cells were kept in a 37°C, 5% CO_2_ humidified incubator and split every 48–72 hours once they reached 100% confluency. New stocks were thawed after cells were passaged 20 times.

### Antibodies

MLCB1, MLCB7, MLCB9, and MLCB11 were provided by Dr. Andrew McGuire (Fred Hutchinson Cancer Research Center). 1G2 and SM5-1 mAbs were produced by GenScript.

### Viral strain and propagation

AD169_GFP_ was a gift from Dr. Donald Coen (Harvard Medical School). This strain encodes an eGFP transgene under control of the HCMV major immediate early promoter (MIEP), which is inserted between US9 and US10 (30). P0 stocks from Dr. Coen were expanded to generate P1 stocks. To mitigate potential mutations from passaging the virus in HFFs, P1 stocks were used to create P2 working stocks. All experiments were performed using these P2 stocks.

P1 and P2 stocks were generated by seeding HFFs in T175 flasks and allowing them to grow to 100% confluency over 48 hours. Infection occurred by adding virus at an MOI of 0.01 in 5 mL of culture medium (DMEM containing 10% FBS, 1% penicillin–streptomycin, and 2 mM L-glutamine). This medium was allowed to sit on the cells for 1 hour at 37°C and was tilted every 15 minutes to ensure even distribution across the bottom of the flask. After the incubation, the virus-containing medium was aspirated; the cells were rinsed with clean medium; and then 25 mL of fresh medium was added.

After the initial infection, the flask was incubated for 14 days at 37°C and periodically checked under the microscope for GFP-positive infected cells. After 100% infection, cells and medium were collected, sonicated, and centrifuged to isolate the virus-containing supernatant. The supernatant was aliquoted and flash-frozen with liquid nitrogen before being transferred into a −80°C freezer. Viral stocks were never refrozen after thawing for use.

To titrate viral stock, we utilized a 50% tissue culture infectious dose assay following the protocol provided by Dr. Felicia Goodrum (University of Arizona). HFFs were seeded at 1 × 10^4^ cells/well into 96-well plates and allowed to grow overnight at 37°C. The viral stock was serially diluted from 10^−1^ to 10^−8^, and 100 μL of each aliquot was added to the plate, such that each row had 12 replicates of one dilution. After 14 days of incubation at 37°C, the wells were fixed with paraformaldehyde (PFA). This was done by washing the wells twice with cold PBS, then incubating them with 4% PFA at room temperature in the dark for 10 minutes, followed by aspiration and two additional washes with cold PBS, leaving the cells in PBS. Each well was visually assessed for GFP and declared GFP positive if two or more cells in the well displayed a GFP signal. The approximate titer value was determined by the total number of GFP-positive wells in each row based on the protocol provided by Dr. Goodrum.

### Checkerboard cocktail neutralization assay

HFFs were seeded on a 96-well plate at 1 × 10^4^ cells/well. The cells were incubated at 37°C for 3 days to ensure 100% confluency. Antibody dilutions were prepared in cell culture medium (DMEM containing 10% FBS, 1% penicillin–streptomycin, and 2 mM L-glutamine) with the following method: a two-dimensional checkerboard titration was performed to assess antibody combinations. Monoclonal antibody 1 (mAb1) was serially diluted across row A of a 96-well deep-well (2 mL) plate, beginning in well A2, where the antibody was prepared at 2× the highest final concentration. Seven total concentrations were generated across the row (A2–A8) using serial dilutions specific to each antibody. In parallel, monoclonal antibody 2 (mAb2) was serially diluted down column 1, beginning in well B1 at 2× the highest final concentration, followed by seven serial dilutions (B1–H1).

To assemble the checkerboard matrix, mAb1 from row A was distributed down each column such that rows A–H contained identical concentrations of mAb1 within each column. Similarly, mAb2 from column 1 was distributed across each row such that columns 1–8 contained identical concentrations of mAb2 within each row. This resulted in a full matrix of pairwise antibody combinations, with each well containing a unique concentration of mAb1 and mAb2. Antibody solutions were combined in equal volumes (1:1) prior to virus addition, so that antibodies were combined at half the concentration of their single-titer counterparts.

Row A and column 1 were reserved as single-antibody controls. Row A contained the serial dilution of mAb1 in the absence of mAb2, while column 1 contained the serial dilution of mAb2 in the absence of mAb1. These controls enabled the generation of individual neutralization curves for each antibody on the same plate as the combination matrix. Once all combinations were distributed to rows B–H and columns 2–8, an equal volume of medium was added to the wells in row A and column 1 to bring the mAbs to a 1× concentration.

Viral inoculum (HCMV strain AD169_GFP_) was diluted to 2× and added to each well in equal volume to the antibody mixtures (1:1), yielding final antibody concentrations of 1× and the desired multiplicity of infection (MOI of 30). This MOI was selected after we tested the fluorescence signal at various time points and MOIs to ensure an optimal dynamic range of the bulk measurement, in a manner identical to our previous study (5). The minimum MOI and time point that resulted in a fivefold increase above background for each strain were chosen for this study. Antibody–virus mixtures were incubated at 37°C for 2 hours to allow for binding and immune complex formation. Three wells per plate were reserved as virus-only controls. After the pre-incubation, the medium from the wells was aspirated, and 100 μL of each mixture was added to a 96-well plate in triplicate.

In the case of a sequential variation of this assay, mAb1 was diluted in a manner described above, and 2× of the virus stock was added directly to mAb1. The single mAb and virus were incubated together for 1 hour at 37°C. After an hour, mAb2 was added to the mAb1:virus mixture, diluted as previously described. The mAb1:mAb2:virus mixture was incubated for an additional hour at 37°C, bringing the total pre-incubation time to 2 hours. After the pre-incubation, the medium from the wells was aspirated, and 100 μL of each mixture was added to a 96-well plate in triplicate.

Once the antibodies and virus were added to the 96-well plate, it was incubated for 24 hours at 37°C. The wells were washed twice with cold PBS, and after aspiration, 4% PFA was added to fix the cells. The plate was then incubated at room temperature in the dark for 10 minutes. When the PFA was aspirated, the plate was washed twice with PBS and left in PBS until it was read via a fluorescent plate reader (BioTek H1) for bulk fluorescent signal. All combinations were performed in technical triplicate.

Percent neutralization was calculated relative to virus-only and uninfected control wells. Data from these experiments were subsequently analyzed using SynergyFinder as described below.

### DiaMOND neutralization assay

HFF seeding, culture conditions, and infection procedures were performed as described above for the neutralization assay. The initial steps of this protocol are very similar to the EMERALD neutralization assay. They are repeated here intentionally to provide a complete, self-contained description of the assay for ease of reference.

For each antibody pair, two independent DiaMOND-style experiments were performed. In the first approach, antibodies were combined by concentration matching so that both mAb1 and mAb2 were mixed at equal concentrations throughout the dilution series. This method was used to match the concentration-based checkerboard assays, which are standard in HCMV research (10, 11, 17). In the second approach, antibodies were combined using dose matching, in which individual antibody titration curves were first used to determine inhibitory concentrations (IC_50_ and IC_90_), and antibodies were then combined at equivalent functional doses (e.g., IC_50_ + IC_50_ or IC_90_+ IC_90_), thereby normalizing for differences in potency between antibodies.

For each condition, antibody dilutions were prepared in complete DMEM (10% FBS, 1% penicillin–streptomycin, and 2 mM L-glutamine). Seven-point serial dilution curves were generated for mAb1 alone and mAb2 alone. In parallel, a seven-point dilution series of the antibody combination was prepared. For concentration-matched experiments, mAb1 and mAb2 were mixed at a 1:1 ratio at each concentration prior to dilution. For dose-matched experiments, mixtures were prepared such that each point in the dilution series corresponded to equivalent inhibitory doses derived from the individual antibody titration curves.

All conditions (mAb1 alone, mAb2 alone, and combination) were performed in technical triplicate. Antibody mixtures were prepared using a modified concentration scheme to accommodate two antibodies. Briefly, 4× stocks of each antibody were generated such that, upon combination, the desired final concentrations would be achieved. For each condition, 4× mAb1 and 4× mAb2 were first combined, and this mixture was then mixed 1:1 with a 2× viral inoculum (HCMV strain AD169_GFP_), yielding final antibody concentrations of 1× and the desired MOI of 30. This approach ensured that both antibodies were combined prior to virus addition, without diluting either component below the intended final concentration. Antibody–virus mixtures were incubated at 37°C for 2 hours to allow for binding and immune complex formation. Three wells per plate were reserved as virus-only controls. After the pre-incubation, the medium from the wells wasaspirated, and 100 μL of each mixture was added to a 96-well plate in triplicate.

Once the antibodies and virus were added to the 96-well plate, it was incubated for 24 hours at 37°C. The wells were washed twice with cold PBS, and after aspiration, 4% PFA was added to fix the cells. The plate was then incubated at room temperature in the dark for 10 minutes. When the PFA was aspirated, the plate was washed twice with PBS and left in PBS until it was read via a fluorescent plate reader (BioTek H1) for bulk fluorescent signal.

Percent neutralization was calculated relative to virus-only and uninfected control wells. Data from these experiments were subsequently analyzed using the Loewe CI framework, as described below.

### EMERALD neutralization assay

HFF seeding, culture conditions, and infection procedures were performed as described above for the neutralization assay. The initial steps of this protocol are very similar to the DiaMOND neutralization assay. Nonetheless, we describe them here to provide a complete, self-contained description of the assay for ease of reference.

For each antibody pair, two complementary titration series were performed. In the first, mAb1 was held constant at its reported IC_50_ concentration, while mAb2 was serially diluted across a seven-point concentration range. In the reciprocal experiment, mAb2 was held constant at its IC_50_, while mAb1 was serially diluted. In parallel, seven-point serial dilution curves were generated for mAb1 and mAb2 individually to serve as single-antibody controls. All conditions were performed in technical triplicate.

Antibody dilutions were prepared in cell culture medium (DMEM containing 10% FBS, 1% penicillin–streptomycin, and 2 mM L-glutamine) using a modified mixing scheme to accommodate two antibodies. Briefly, 4× stocks of each antibody were prepared such that, upon combination, the desired final concentrations would be achieved. For each condition, 4× mAb1 and 4× mAb2 were first combined, and this mixture was then mixed 1:1 with a 2× virus stock to yield final 1× concentrations of each antibody and virus. This 4× scheme was used to ensure that both antibodies could be combined prior to virus addition without diluting below the intended final concentration.

Antibody–virus mixtures were pre-incubated at 37°C for 2 hours to allow complex formation. Three wells per plate were reserved as virus-only controls. Following pre-incubation, medium was aspirated from the HFF monolayers, and 100 μL of each antibody–virus mixture was added to the plate in triplicate. Cells were incubated at 37°C with 5% CO_2_ for 24 hours. The wells were washed twice with cold PBS, and after aspiration, 4% PFA was added to fix the cells. The plate was then incubated at room temperature in the dark for 10 minutes. When the PFA was aspirated, the plate was washed twice with PBS and left in PBS until it was read via a fluorescent plate reader (BioTek H1) for bulk fluorescent signal.

Percent neutralization was calculated relative to virus-only and uninfected control wells. Data from these experiments were subsequently analyzed using the Loewe CI framework as described below.

### Synergy analysis using SynergyFinder 3.0

After measuring the relative fluorescent units, the percentage of inhibition was calculated as neutralization (%) = (max − value) /(max − min) × 100, where max is the mean of “no mAb” or “virus alone” wells, and min is the mean of the negative control (uninfected) wells. All combinations were performed in technical triplicate, and the data were averaged for analysis.

The neutralization (%) was inputted into SynergyFinder 3.0 in matrix format, with rows and columns corresponding to the concentrations of mAb1 and mAb2, respectively. Combination effects were evaluated using the Loewe additivity reference model. Observed neutralization values were compared to the expected additive response calculated by the Loewe model across the full concentration matrix. Synergy scores were computed for each dose combination as the deviation between observed and expected responses.

SynergyFinder-generated outputs included synergy score heatmaps and three-dimensional response surface plots. Positive synergy scores indicated synergistic interactions (greater-than-additive effects); values near 0 indicated additive interactions, and negative scores indicated antagonism (less-than-additive effects). Summary synergy scores were calculated by averaging across the entire matrix, providing an overall measure of interaction for each antibody pair.

### Loewe CI calculation and analysis for DiaMOND

To quantify antibody interactions in the DiaMOND neutralization assays, combination effects were analyzed using a Loewe CI approach in Microsoft Excel. This analysis compares the amount of antibody used in the combination to the amount of each antibody required to achieve the same level of neutralization when used alone. For each point in the antibody combination titration, the observed neutralization value was defined as *E*. For example, if a combination well produced 65% neutralization, then *E* = 65. This observed combination effect was then compared back to the single-antibody titration curves for mAb1 and mAb2.

For each antibody, the single-antibody titration curve was used to identify the two measured neutralization values that surrounded *E*. The lower value was defined as *E*_low_, and the higher value was defined as *E*_high_. For example, if *E* = 65% and the mAb1-alone curve contained measured neutralization values of 50% and 70%, then *E*_low_ = 50% and *E*_high_ = 70%*.*

The corresponding antibody concentrations at those two points were defined as *C*_low_ and *C*_high_*.* In this example, if 50% neutralization occurred at 1 µg/mL and 70% neutralization occurred at 3 µg/mL, then *C*_low_ = *1* and *C*_high_ = 3*.*

Because the single-antibody curve did not necessarily contain a measured point at exactly 65% neutralization, the concentration required to produce *E* was estimated by linear interpolation. In this context, *D_x_* represents the interpolated concentration of a single antibody required to achieve the observed level of neutralization *E*. This value is derived from the single-antibody titration curve using the bounding values *E*_low_ and *E*_high_ and their corresponding concentrations *C*_low_ and *C*_high_, as described above:


$$D_{x}=C_{low}+\left(\frac{E-E_{low}}{E_{high}-E_{low}}\right)\times (C_{high}-C_{low}).$$

This interpolation was performed independently for each antibody to generate *D*_1_*_X_* and *D*_2_*_X_*, which represent the amounts of mAb1 and mAb2, respectively, required to produce the observed combination effect if each antibody were acting alone. In contrast, *D*_1_ and *D*_2_ represent the actual concentrations of mAb1 and mAb2 present in the combination well, as defined by the experimental setup. For example, for a 1:1 mixture, each antibody contributes half of the total concentration, so *f*_mAb1_ = *f*_mAb2_ = 0.5. Therefore, *D*_1_ = *f*_mAb1_ × *D*_total_, *D*_2_ = *f*_mAb2_ × *D*_total_ .

For dose-matched DiaMOND experiments, antibody ratios were determined based on equivalent inhibitory doses (e.g., IC_50_ or IC_90_) derived from individual titration curves. The fractional contributions of each antibody were therefore defined as$f_{mAb1}=\frac{D_{1}}{D_{1}+D_{2}},\;f_{mAb2}=\frac{D_{2}}{D_{1}+D_{2}}$, where *D*_1_ *a*nd *D*_2_ represent the concentrations of each antibody in the mixture. Fractional contributions were defined by the fixed mixing ratio and remained constant across the dilution series.

In this context, the fractional contributions are used to construct the fixed-ratio mixtures by partitioning the total antibody concentration into defined amounts of mAb1 and mAb2 at each point in the dilution series. These fractions, therefore, determine how the total dose is distributed between the two antibodies and aid in calculating *D*_1_ and *D*_2_ but are not used directly in the subsequent Loewe CI calculation.

The Loewe CI was then calculated for each mixture datapoint as: $CI=\frac{D_{1}}{D_{2A}}+\frac{D_{2}}{D_{2A}}$. CI values were interpreted as follows: CI < 0.9, synergistic interaction; 0.9 < CI < 1.1, additive interaction; and CI > 1.1, antagonistic interaction, as determined by experimental error between replicates. To reduce noise from low-effect regions and experimental variability, only data points within the assay’s dynamic range (i.e., excluding near-zero or plateau effects) were included in the summary statistics. Mean and median CI values were calculated across all valid data points for each antibody pair to provide an overall measure of interaction.

### Loewe CI calculation and analysis for EMERALD

To quantify antibody interactions in the EMERALD neutralization assays, combination effects were analyzed using a Loewe CI approach in Microsoft Excel. This analysis compares the amount of antibody used in the combination to the amount of each antibody required to achieve the same level of neutralization when used alone. In contrast to DiaMOND experiments, EMERALD assays were performed by holding one antibody constant at its IC_50_ while titrating the second antibody across a range of concentrations.

For each point in the antibody combination titration, the observed neutralization value was defined as *E*. For example, if a combination well produced 65% neutralization, then *E* = 65*.* This observed combination effect was then compared back to the single-antibody titration curves for mAb1 and mAb2.

For each antibody, the single-antibody titration curve was used to identify the two measured neutralization values that surrounded *E*. The lower value was defined as E_low_, and the higher value was defined as *E*_high_. For example, if *E* = 65% and the mAb1-alone curve contained measured neutralization values of 50% and 70%, then *E*_low_ = 50% and *E*_high_ = 70%.

The corresponding antibody concentrations at those two points were defined as *C*_low_ and *C*_high_. In this example, if 50% neutralization occurred at 1 µg/mL and 70% neutralization occurred at 3 µg/mL, then *C*_low_ = *1* and *C*_high_ = 3*.*

Because the single-antibody curve did not necessarily contain a measured point at exactly 65% neutralization, the concentration required to produce *E* was estimated by linear interpolation. In this context, *D_x_* represents the interpolated concentration of a single antibody required to achieve the observed level of neutralization *E*. This value is derived from the single-antibody titration curve using the bounding values *E*_low_ and *E*_high_ and their corresponding concentrations *C*_low_ and *C*_high_, as described above: $D_{x}=C_{low}+\left(\frac{E-E_{low}}{E_{high}-E_{low}}\right)\times (C_{high}-C_{low})$. This interpolation was performed independently for each antibody to generate *D*_1_*_X_* and *D*_2_*_X_*, which represent the amounts of mAb1 and mAb2, respectively, required to produce the observed combination effect if each antibody were acting alone.

In contrast, *D*_1_ and *D*_2_ represent the actual concentrations of mAb1 and mAb2 present in the combination well, as defined by the EMERALD experimental design. Specifically, one antibody was held constant across the entire titration series at its IC_50_, while the second antibody was serially diluted. Therefore, for each data point, one of *D*_1_ or *D*_2_ is fixed, while the other varies across the dilution series.

The Loewe CI was then calculated for each mixture datapoint as: $CI=\frac{D_{1}}{D_{2A}}+\frac{D_{2}}{D_{2A}}$. CI values were interpreted as follows: CI > 1.1, synergistic interaction; 0.9 < CI < 1.1, additive interaction; and CI > 1.1, antagonistic interaction, as determined by experimental error between replicates. To reduce noise from low-effect regions and experimental variability, only data points within the assay’s dynamic range (i.e., excluding near-zero or plateau effects) were included in the summary statistics. Mean and median CI values were calculated across all valid data points for each antibody pair to provide an overall measure of interaction.

EMERALD experiments are defined directly in terms of antibody concentrations rather than dose units, such that *D*_1_ and *D*_2_ are explicitly specified for each condition. As a result, it is not necessary to calculate fractional contributions (*f*_mAb1_ and *f*_mAb2_) to construct the mixtures, as was done in the DiaMOND framework.

## Data Availability

All data generated in this study are included in the main text or supplemental material.

## SUPPLEMENTAL MATERIAL

Supplemental material jvi.00879-26-s0001.pdf Fig. S1 to S4; Table S1.

ASM does not own the copyrights to Supplemental Material that may be linked to, or accessed through, an article. The authors have granted ASM a non-exclusive, world-wide license to publish the Supplemental Material files. Please contact the corresponding author directly for reuse.
