# Supplementary material for: Comparative evaluation of screening approaches for antiviral monoclonal antibody combinations
Source: J Virol. 2026 Sep 2;100(9):e00879-26. doi: 10.1128/jvi.00879-26 (PMC13595932; doi:10.1128/jvi.00879-26)
Supplement: Supplementary file 1 [file jvi.00879-26-s0001.pdf]

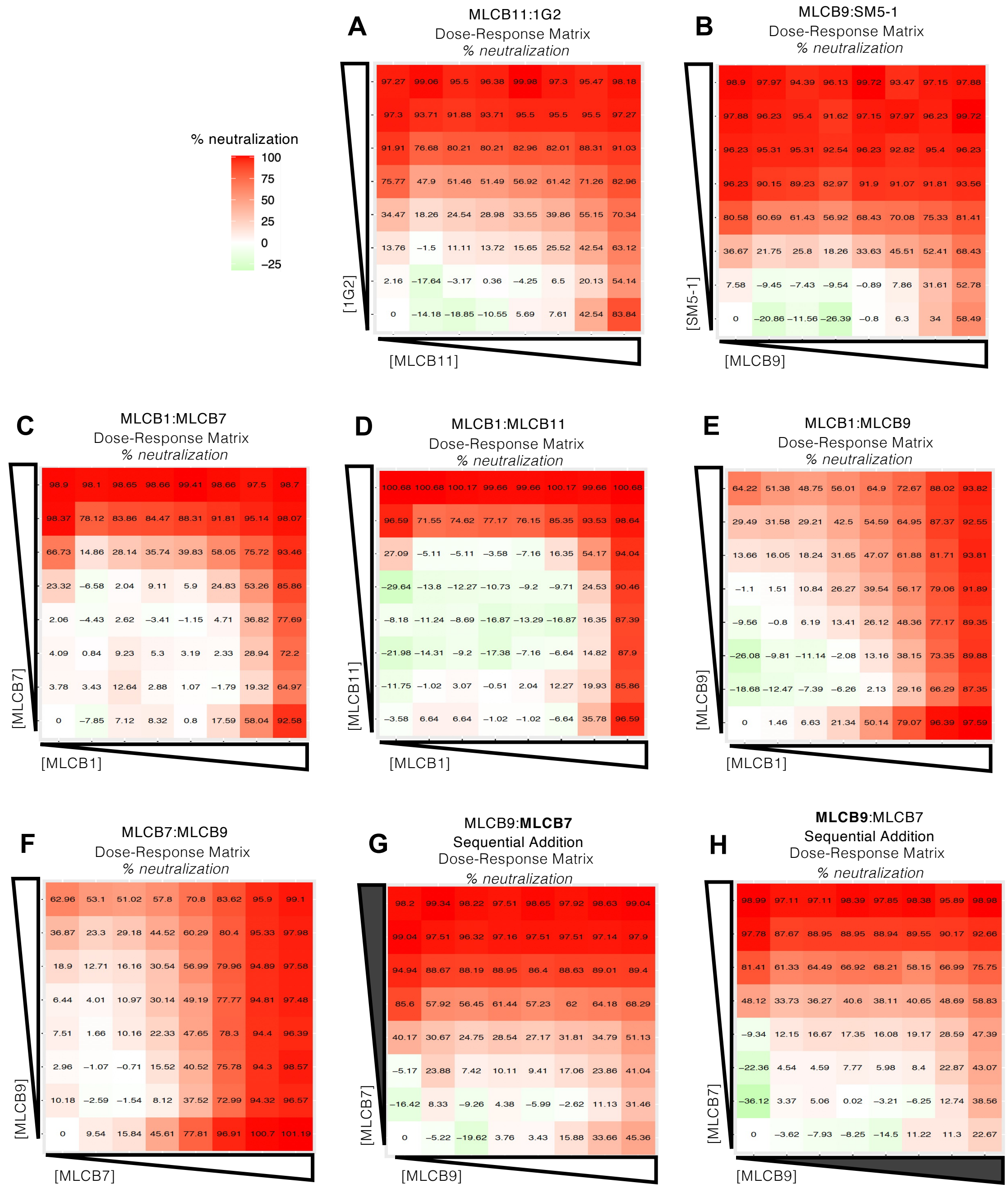

Figure S1. Checkerboard assay dose-response matrices for mAb combination neutralization assays against HCMV AD169$_{GFP}$. mAbs were tested in combination in checkerboard viral neutralization assays against HCMV AD169$_{GFP}$. Antibodies were combined at a 1:1 ratio and tested across matched concentration matrices (0–25 µg/mL). Heatmaps display percent neutralization (% inhibition) across the full checkerboard matrix for each antibody combination. The mAb listed first is represented on the x-axis, and the mAb listed second is represented on the y-axis. Triangular wedges along the axes indicate increasing concentrations of each antibody across the checkerboard matrix. Results are shown as the average of three plate replicates. The results correspond to the data shown in Figures 2-4. (A) (left) Percent neutralization scale corresponding to the following graphs, with red indicating more neutralization. (right) MLCB11 and 1G2. (B) MLCB9 and SM5-1. (C) MLCB1 and MLCB7. (D) MLCB1 and MLCB11. (E) MLCB1 and MLCB9. (F) MLCB7 and MLCB9. (G-H) Sequential checkerboard viral neutralization assays in which mAbs were added sequentially prior to infection. The antibody added first is indicated in bolded text and with a filled-in triangular wedge. For sequential assays, MLCB9 is represented on the x-axis and MLCB7 is represented on the y-axis. (G) MLCB7 was added prior to MLCB9. (H) MLCB9 was added prior to MLCB7.

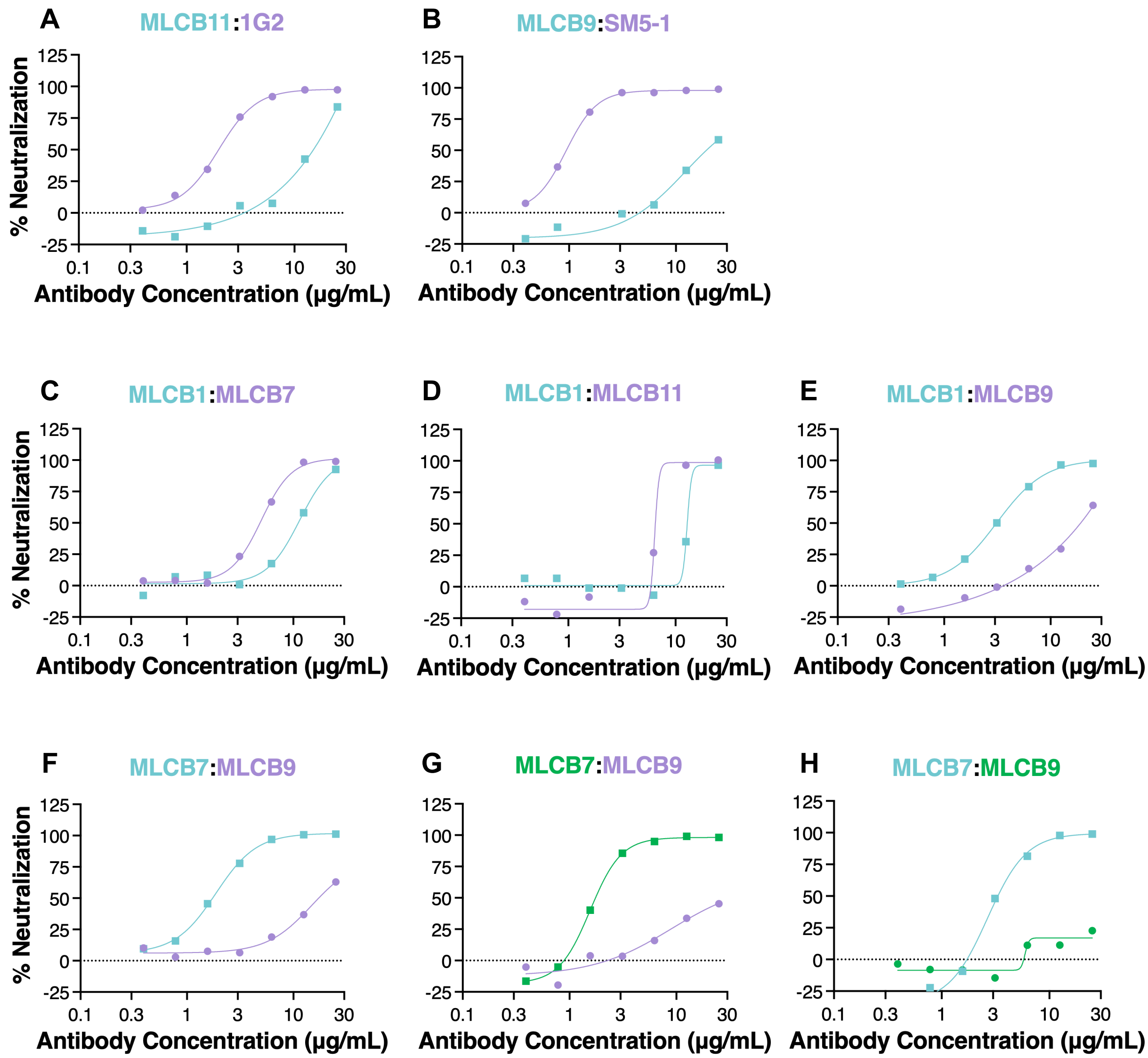

Figure S2. Individual monoclonal antibody neutralization curves corresponding to checkerboard assay combinations. Individual monoclonal antibody (mAb) neutralization curves corresponding to the checkerboard assay combinations shown in Figures 2–4. mAbs were tested individually in viral neutralization assays against HCMV AD169$_{GFP}$ across a concentration range of 0–25 µg/mL. Percent neutralization (% inhibition) is plotted as a function of antibody concentration (µg/mL). The first mAb listed in each panel is shown in light blue and the second mAb is shown in purple. For sequential neutralization assays, the antibody added first is indicated in green. Data points represent the average of three plate replicates, and curves were generated using nonlinear regression analysis in GraphPad Prism. Hill slopes for each curve are reported in brackets after the mAb name. (A) MLCB11 [0.823] and 1G2 [1.786]. (B) MLCB9 [0.495] and SM5-1 [2.423]. (C) MLCB1 [1.568] and MLCB7 [2.161]. (D) MLCB1 [1.803] and MLCB11 [1.091]. (E) MLCB1 [2.407] and MLCB9 [1.303]. (F) MLCB7 [2.053] and MLCB9 [1.197]. (G) Sequential neutralization assay in which MLCB7 [2.986] was added prior to MLCB9 [1.184]. (H) Sequential neutralization assay in which MLCB9 [24.39] was added prior to MLCB7 [2.419].

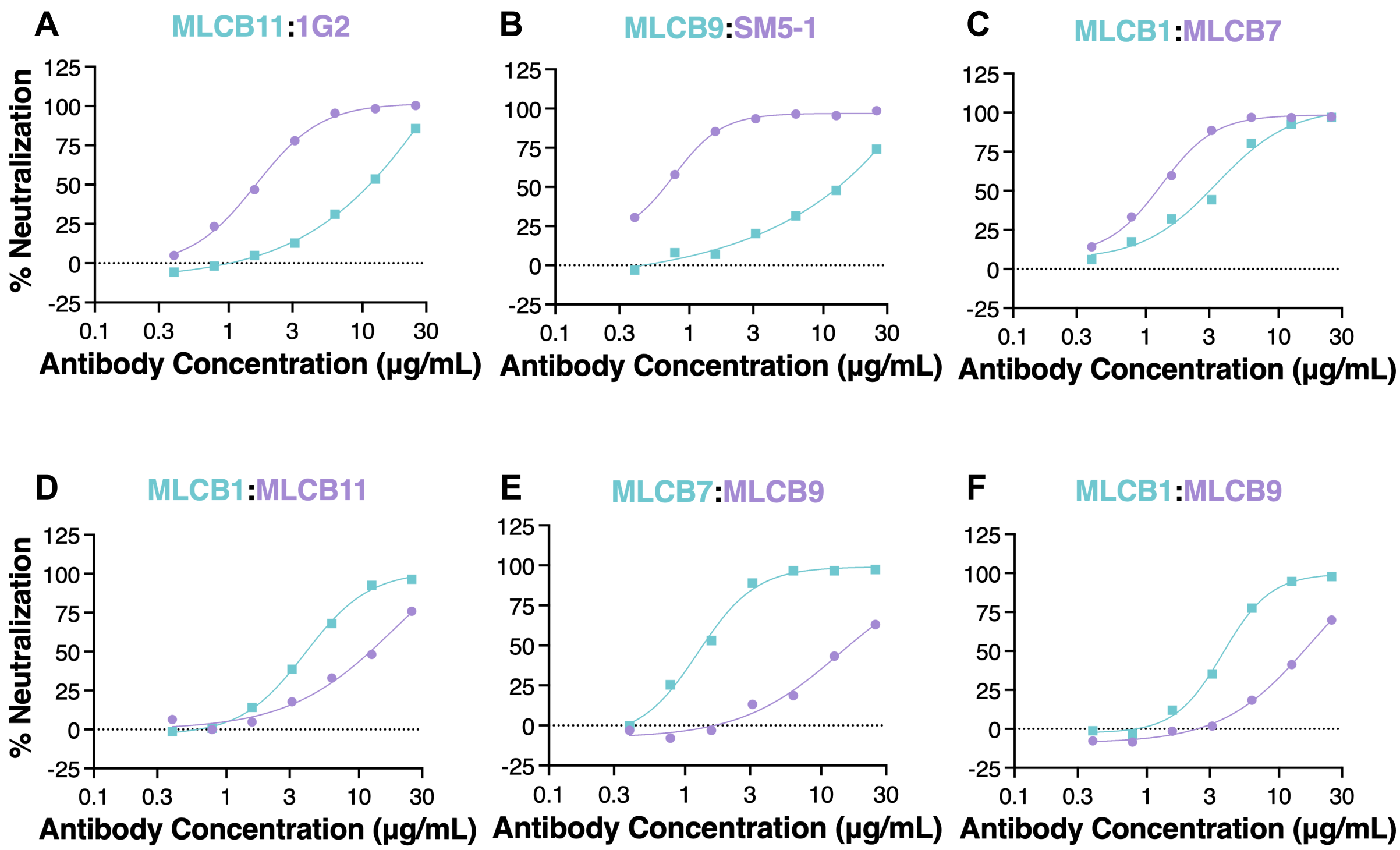

Figure S3. Individual monoclonal antibody neutralization curves corresponding to concentration-based DiaMOND assays and EMERALD assays. Individual monoclonal antibody (mAb) neutralization curves corresponding to the concentration-based DiaMOND assays shown in Figure 5 and EMERALD assays shown in Figure 7. mAbs were tested individually in viral neutralization assays against HCMV $AD169_{GFP}$ across a concentration range of 0–25 µg/mL. Percent neutralization (% inhibition) is plotted as a function of antibody concentration (µg/mL). The first mAb listed in each panel is shown in light blue, and the second mAb is shown in purple. Data points represent the average of three plate replicates, and curves were generated using nonlinear regression analysis in GraphPad Prism. Hill slopes for each curve are reported in brackets after the mAb name. (A) MLCB11 [0.823] and 1G2 [1.786]. (B) MLCB9 [0.495] and SM5-1 [2.423]. (C) MLCB1 [1.568] and MLCB7 [2.161]. (D) MLCB1 [1.803] and MLCB11 [1.091]. (E) MLCB7 [2.053] and MLCB9 [1.197]. (F) MLCB1 [2.407] and MLCB9 [1.303].

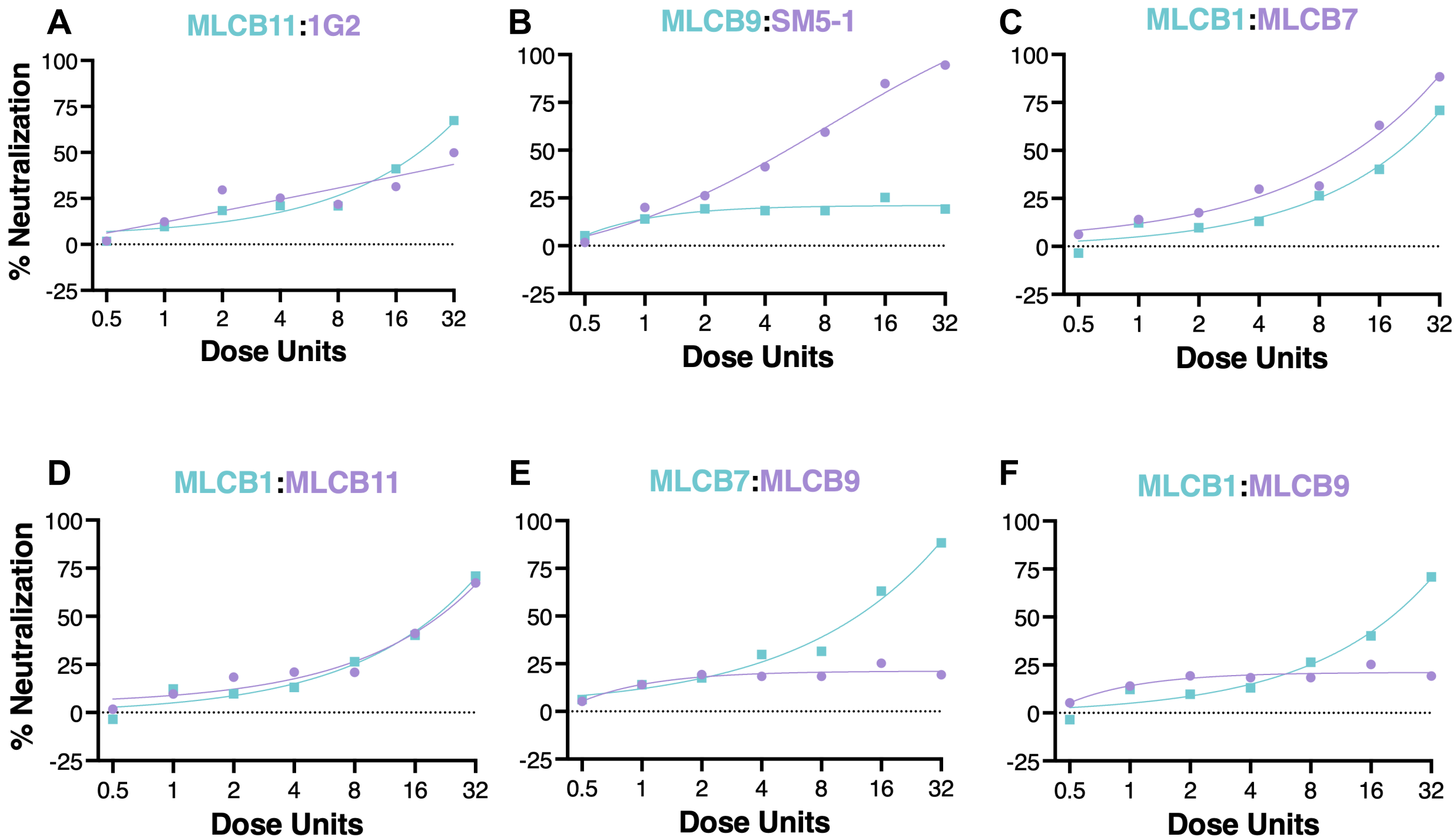

Figure S4. Individual monoclonal antibody neutralization curves corresponding to dose-based DiaMOND assays. Individual monoclonal antibody (mAb) neutralization curves corresponding to the dose-based DiaMOND assays shown in Figure 6. mAbs were tested individually in viral neutralization assays against HCMV $AD169_{GFP}$ across doses as calculated by individual titration curves. Percent neutralization (% inhibition) is plotted as a function of antibody dose. The first mAb listed in each panel is shown in light blue, and the second mAb is shown in purple. Data points represent the average of three plate replicates, and curves were generated using nonlinear regression analysis in GraphPad Prism. Hill slopes for each curve are reported in brackets after the mAb name. (A) MLCB11 [0.737] and 1G2 [0.035]. (B) MLCB9 [1.183] and SM5-1 [0.713]. (C) MLCB1 [0.726] and MLCB7 [0.636]. (D) MLCB1 [0.726] and MLCB11 [0.737]. (E) MLCB7 [0.636] and MLCB9 [1.183]. (F) MLCB1 [0.726] and MLCB9 [1.183].

mAb	IC_{50}, $AD169_{GFP}$ (μg/ml)
1G2	0.33 +/- 0.02
SM5-1	0.32 +/- 0.01
MLCB1	0.28 +/- 0.09
MLCB7	1.09 +/- 0.18
MLCB9	4.89 +/- 0.31
MLCB11	3.02 +/- 1.14

Supplemental Table S1: IC_{50} values for mAbs used in this study against $AD169_{GFP}$. IC_{50} values and SEMs were previously published for each mAb against $AD169_{GFP}$ in McClave et al. 2026 (Reference 5, Table 1). Titration curves were established from neutralization assays performed in technical and biological triplicate. IC_{50} values were interpolated from nonlinear regression analysis of the titration curves in GraphPad Prism.
